# The drug efficacy testing in 3D cultures platform identifies effective drugs for ovarian cancer patients

**DOI:** 10.1038/s41698-023-00463-z

**Published:** 2023-10-31

**Authors:** Emma Åkerlund, Greta Gudoityte, Elisabeth Moussaud-Lamodière, Olina Lind, Henri Colyn Bwanika, Kaisa Lehti, Sahar Salehi, Joseph Carlson, Emelie Wallin, Josefin Fernebro, Päivi Östling, Olli Kallioniemi, Ulrika Joneborg, Brinton Seashore-Ludlow

**Affiliations:** 1 Science for Life Laboratory, Department of Oncology-Pathology, Karolinska Institute, Stockholm, Sweden; 2 Department of Oncology-Pathology, Karolinska Institute, Stockholm, Sweden; 3 Department of Biomedical Laboratory Science, Norwegian University of Science and Technology NTNU, Trondheim, Norway; 4 Department of Microbiology, Tumor and Cell Biology, Karolinska Institutet, Stockholm, Sweden; 5 Department of Pelvic Cancer, Theme Cancer, Karolinska University Hospital, Stockholm, Sweden; 6 Department of Women’s and Children’s Health, Division of Obstetrics and Gynecology, Karolinska Institutet, Stockholm, Sweden; 7 Department of Pathology and Laboratory Medicine, Keck School of Medicine, University of Southern California, Los Angeles, CA 90089 USA; 8 Institute for Molecular Medicine Finland, University of Helsinki, Helsinki, Finland; 9 Present Address: Department of Oncology-Pathology, Karolinska Institute, Stockholm, Sweden; 10 Present Address: Department of Pelvic Cancer, Theme Cancer, Karolinska University Hospital, Stockholm, Sweden; 11 Present Address: Department of Women’s and Children’s Health, Division of Obstetrics and Gynecology, Karolinska Institutet, Stockholm, Sweden

**Keywords:** Ovarian cancer, Ovarian cancer

## Abstract

Most patients with advanced ovarian cancer (OC) relapse and progress despite systemic therapy, pointing to the need for improved and tailored therapy options. Functional precision medicine can help to identify effective therapies for individual patients in a clinically relevant timeframe. Here, we present a scalable functional precision medicine platform: DET3Ct (Drug Efficacy Testing in 3D Cultures), where the response of patient cells to drugs and drug combinations are quantified with live-cell imaging. We demonstrate the delivery of individual drug sensitivity profiles in 20 samples from 16 patients with ovarian cancer in both 2D and 3D culture formats, achieving over 90% success rate in providing results six days after operation. In this cohort all patients received carboplatin. The carboplatin sensitivity scores were significantly different for patients with a progression free interval (PFI) less than or equal to 12 months and those with more than 12 months (*p* < 0.05). We find that the 3D culture format better retains proliferation and characteristics of the in vivo setting. Using the DET3Ct platform we evaluate 27 tailored combinations with results available 10 days after operation. Notably, carboplatin and A-1331852 (Bcl-xL inhibitor) showed an additive effect in four of eight OC samples tested, while afatinib and A-1331852 led to synergy in five of seven OC models. In conclusion, our 3D DET3Ct platform can rapidly define potential, clinically relevant data on efficacy of existing drugs in OC for precision medicine purposes, as well as provide insights on emerging drugs and drug combinations that warrant testing in clinical trials.

## Introduction

Precision medicine aims to match the right drug to the right patient at the right time to avoid toxic or ineffective treatment regimens. Real-time molecular characterization of each patient at the genetic and transcriptomic levels has pioneered implementation of precision medicine practices in the clinic, especially in cancer treatment. Using this approach, the presence of driver mutations or copy number alterations is used to stratify patients to a matching targeted therapy. However, the overall benefit of these efforts has been varying and there is a major opportunity to improve personalized therapy allocation^1,2^.

Functional precision medicine (*f*PM) approaches can complement current treatment allocation based on molecular profiles. In *f*PM the response to drugs is evaluated ex vivo in patient cells to identify effective drugs for each individual^1^. Successful implementation of *f*PM-based treatment allocation has been demonstrated in previous studies with the majority addressing hematological malignancies^3–5^. However, to date there are few reports describing the use of *f*PM to tailor combination treatments for individual patients^6^ and the application of these techniques to solid tumors has advanced more slowly, perhaps due to the challenges reviewed by Letai and colleagues^7^. Instead, recent approaches to drug testing in solid tumors have included patient-derived xenograft (PDX), patient-derived cell (PDC), and patient-derived organoid (PDO) models^8,9^. PDO models have been shown to recapitulate patient response, providing excellent tools for the discovery of new therapeutics or drug repurposing studies. However, deriving these types of models takes weeks or months and current methods are far from ensuring success for all samples^10,11^. In fact, these two challenges impede implementation of these approaches in the clinic, as they would yield unacceptable treatment delays. Accordingly, methods enabling rapid drug testing in patient cells in models reflecting the individual pathobiology are needed to impact treatment decisions.

Here, we describe a scalable *f*PM platform meeting these challenges for the rapid generation of patient-specific drug sensitivity and resistance profiles applicable to solid tumors, called Drug Efficacy Testing in 3D Cultures (DET3Ct). To circumvent the lengthy process of model development we exploit fresh uncultured cells for ex vivo drug testing. These complex samples contain not only cancer cells, but also the associated cells from the microenvironment. We demonstrate this platform in ovarian cancer, a disease where treatment allocation is mainly based on stage and status of the patient rather than individual pathobiology.

Despite the heterogeneous genomic landscape and the plethora of different histotypes/histologic subtypes of ovarian cancer, standard treatment is cytoreductive surgery and platinum-based chemotherapy^12,13^. Furthermore, even with optimized surgical strategies and the introduction of new therapies, such as poly ADP polymerase (PARP) inhibitors or antiangiogenic agents, the long-term survival in OC has not improved significantly during the last decades^14^. Thus, while most patients with OC respond well to initial treatment, they relapse within the first two years with few effective treatment options available^15^. This highlights the urgent need for deeper understanding of the specific pathobiology of each patient and for identification of more individualized treatment strategies.

Using the DET3Ct platform we generate patient-specific drug sensitivity profiles within six days, a timeframe compatible with clinical decision timelines. Based on these results we rationally design and evaluate patient-specific drug combinations within 10 days. Our studies reveal that the drug sensitivity scores from our platform for carboplatin and cisplatin can discriminate between patients with short (≤12 months) and long (>12 months) progression-free intervals. Interestingly, using our drug combination platform we uncover an effective combination of the tyrosine kinase inhibitor, afatinib, and the Bcl-xL inhibitor, A-1331852, for the treatment of OC. We observed a synergistic interaction between these compounds across 3D cultures of ovarian cancer cell lines and additional patient-derived models, which was not observed in patient-derived fibroblast models. We explore the molecular underpinnings of the observed synergy and uncover both Bcl-xL and BIM upregulation through EGFR inhibition in ovarian cancer cells. Ultimately, based on the results presented here, we foresee that the DET3Ct platform can be utilized for drug positioning to find better therapeutic options for patients suffering from OC and extended to other solid tumor types for improved treatment decisions.

## Results

### The DET3Ct platform identifies drug sensitivity in patient-derived cells cultured in 3D

**Fig. 1:**
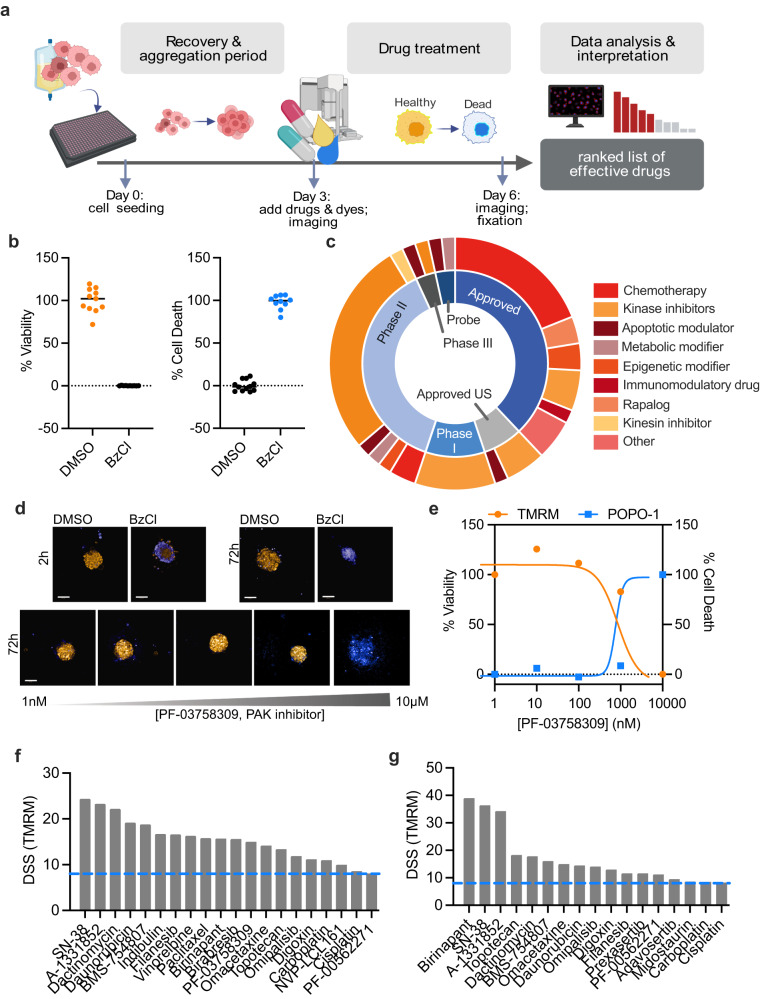
Optimization of the DET3Ct platform for the identification of effective drugs in PDCs. **a** Schematic overview of the assay design and objectives (figure created with BioRender.com). **b** Plots displaying the normalized measurements for OvCa027 for negative (DMSO, *n* = 11) and positive (BzCl, *n* = 10) controls. Lines at median. Live cells are denoted by high TMRM (yellow) and dead cells by high POPO-1 (blue). **c** An overview of the phase and compound class of the 58 drugs in the OC drug repurposing library. **d** Example of maximum projection images after drug exposure to PF-03758309 for 72 h in a five-point concentration range, as well as the corresponding controls at time 2 h and 72 h. The white scale bar represents 100 μm. Blue is POPO-1 iodide and orange is TMRM. **e** Corresponding concentration-response curves of the data in (**d**). The DSS score for TMRM is 5.4 and 2.7 for POPO-1. **f** Waterfall plot of the DSS calculated using the TMRM parameter for effective drugs (DSS > 8) in the OvCa024 PDCs after 72 h treatment with the OC repurposing library. **g** Waterfall plot of the DSS calculated using the TMRM parameter for effective drugs (DSS > 8) in the OvCa027 PDCs after 72 h treatment with the OC repurposing library.

Here we set out to establish a platform to provide individual drug sensitivity profiles for ovarian cancer patients that is compatible with treatment timeframes. In anticipation of the complexity associated with assaying heterogenous primary cultures, we opted to develop a live-cell imaging assay quantifying cell health and death. A three-day recovery period after sample processing and prior to addition of dyes and drugs was built into the protocol to allow for reaggregation of tissue or ascites (Fig. 1a). During this time, cells self-assemble into spheroids or aggregates. These are imaged upon the addition of drugs to ensure retained viability and then again 72 h after treatment to evaluate ex vivo drug response. Initial optimization of combinations of live-cell dyes was done on stable established primary cell cultures models, PDCs OvCa024 and OvCa027, generated in our lab (Supplementary Table 1). Optimization studies suggested that a combination of tetramethylrhodamine methyl ester (TMRM) and POPO-1 iodide, measuring mitochondrial polarization, and cytoplasmic membrane permeabilization, respectively, demonstrated robust quantification of cell health and death in the image-based assay with no measured effect on cell proliferation. We then established an in-house image analysis pipeline to evaluate cell viability. A satisfactory assay window was observed for cell health based on the ratio of TMRM volume to the composite volume calculated from all wavelengths, as well as the cell death ratio of POPO-1 volume to Hoechst33342 nuclei volume, when comparing positive and negative controls (Fig. 1b). To further evaluate our assay concept, we designed a drug library consisting of 58 different small molecules relevant to ovarian cancer treatment covering a five-point concentration range for each drug, referred to here as the OC repurposing library (Fig. 1c, for full description of the OC library see Supplementary Table 2). Using our optimized conditions, we screened OvCa027 and OvCa024 against the OC repurposing library. For both samples, satisfactory Z’ scores were observed for TMRM (0.59 for OvCa024, 0.59 for OvCa027) and POPO-1 (0.48 for OvCa024 and 0.57 for OvCa027) parameters, which indicate reproducibility of the assay. The results from the image analysis pipeline were imported into the Breeze web-based application, where well-based quantification of cell health and death was converted to concentration-response curves and the drug sensitivity score (DSS) for each drug for both TMRM and POPO-1 (Fig. 1d, e). For the two models we observed 20 hits (DSS > 8) for OvCa024 and 17 hits for OvCa027 (Fig. 1f, g, Supplementary Table 3). Among the top most effective compounds were carboplatin and paclitaxel, which are the standard of care therapy for ovarian cancer. In addition, both models were sensitive to the Bcl-xL inhibitor, A-1331852 and the topoisomerase I inhibitor, SN-38. In the OvCa027 PDC model far fewer hits for the POPO-1 parameter than TMRM were observed (Supplementary Fig. 1a–c). A potential reason for this is that the pharmacokinetics of cell death vary. For some drugs we observe relatively intact cell structures, whereas in other cases cells had already condensed, or completely disaggregated. This adds an additional challenge in interpretation with the POPO-1 readout and in further analyses we therefore focus mainly on the TMRM parameter.

### Carboplatin drug sensitivity scores from the DET3Ct platform associate with clinical response

We next applied the DET3Ct platform to primary uncultured patient samples. To assess the feasibility and reproducibility of the method, we characterized the drug sensitivity of 20 samples from 16 patients against the OC repurposing library. Primary material was acquired from consenting patients in conjunction with surgery. The majority of the patient samples were from high grade serous (HGSOC) OC with a few samples from rare subtypes, such as low grade serous (LGSOC) or mucinous (MOC) OC (Table 1, Supplementary Table 4). Most patients were diagnosed at International Federation of Gynecology and Obstetrics (FIGO) stage 3 or 4, two patients had recurrent disease, and one patient had received neoadjuvant chemotherapy prior to surgery. One patient (040 A) was later diagnosed with struma ovarii accompanied by thyroid papillary carcinoma. This patient received radio-active iodine treatment, and is excluded from downstream response prediction analyses. Two patients were lost to further follow-up after surgery.

**Table 1:** Clincial information of the patient material passing quality control used in this study.

Case ID	Material assayed	Diagnosis-histology	Stage	Ca-125
OvCa_025	Tissue	HGS peritoneal cancer	2B	81
OvCa_026	Tissue	HGS endometrial cancer	4B	2560
OvCa_030	Tissue	HGS tubal cancer	3 C	2960
OvCa_031	Tissue	HGSOC	4 A	3130
OvCa_037	Tissue	HGSOC	3 C	1620
OvCa_038	Tissue	HGS tubal cancer	3 C	125
OvCa_039	Ascites	HGSOC	3 C	103
OvCa_040	Ascites	Struma ovarii with papillary thyroid cancer	1 C	1720
OvCa_041	Ascites	HGSOC	3 C	864
OvCa_042	Tissue/ascites	Recurrent MUCOC from 2018	1 A (2018)	356
OvCa_043	Ascites	HGS tubal cancer	3 C	531
OvCa_044	Tissue	LGSOC	3 C	99
OvCa_045	Tissue/ascites	Recurrent LGSOC from 1991	3 A (1991)	318
OvCa_047	Tissue/ascites	HGS tubal cancer	4B	2320
OvCa_050	Tissue/ascites	HGS tubal cancer	3 C	1580
OvCa_053	Ascites	HGS tubal cancer	3 C	498

**Fig. 2:**
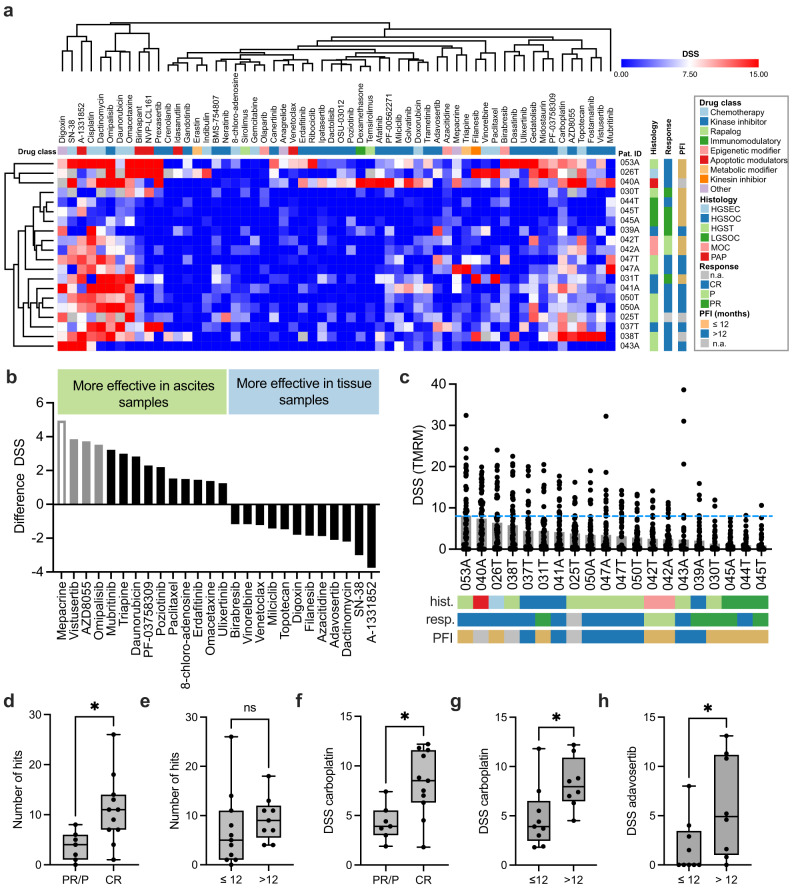
The DET3Ct platform reports patient-specific drug response in 6 days. **a** An overview of the drug response landscape presented in a heatmap showing the DSS scores from the TMRM parameter. Patient samples are listed on the x-axis where A stands for sample coming from ascites and T for tissue. Drugs are shown on the y-axis. Clustering was performed in Morpheus^51^ using Euclidean distance and complete linkage. The key in the figure delineates drug class, patient disease histology, clinical response as measured by RECIST, and PFI in months. **b** Differences in DSS between the four paired ascites and tissue samples. Grey bars indicate compounds known to act through the PI3k/mTOR pathways. Grey bars with no fill represents compounds likely acting through PI3K/mTOR pathways. **c** Barplot showing the median DSS score for each patient. Each dot represents one drug from the OC Repurposing library. The dotted blue line denotes DSS 8, the cut-off defining drug sensitivity. The keys to the patient information are found in panel (**a**). **d** Boxplot of the number of effective drugs for patient classes according to RECIST guidelines, PR/P (*n* = 5) and CR (*n* = 11). Two-tailed Mann–Whitney *U* test *p* = 0.0107. **e** Boxplot of the number of effective drugs for patients with PFI ≤ 12 months (*n* = 7) and PFI > 12 months (*n* = 7). Two-tailed Mann–Whitney *U* test *p* = 0.1931. **f** Boxplot of the carboplatin DSS for patients with PR/P (*n* = 5) and CR (*n* = 11) RECIST classes. Two-tailed Mann–Whitney *U* test *p* = 0.0114. **g** Boxplot of the carboplatin DSS for patients with PFI ≤ 12 months (*n* = 7) and PFI > 12 months (*n* = 7). Two-tailed Mann–Whitney *U* test *p* = 0.0206. **h** Bar plot of the adavosertib DSS for patients with PFI ≤ 12 months (*n* = 7) and PFI > 12 months (*n* = 7). Two-tailed Mann–Whitney *U* test *p* = 0.0453. Boxplots show minimum, maximum and all points.

Tissue and ascites were immediately processed and tested for drug sensitivity to the OC repurposing library. Using the DET3Ct platform we could report a patient-specific drug sensitivity profile, consisting of a ranked list of effective drugs in the OC repurposing library within six days. As expected, cell growth and morphology of the samples varied across the cohort (Supplementary Fig. 2a). Despite this variation, 20 of 22 samples passed our assay requirement of Z’ > 0.4 for the TMRM parameter resulting in total 1143 DSSs after quality control of the concentration-response curves (Fig. 2a, Supplementary Fig. 2b, c, Supplementary Table 5). In the primary uncultured samples, the POPO-1 parameter varied more greatly across samples. A Z’ > 0.4 was observed for 15 samples (Supplementary Fig. 2d, e). Based on this, we opted to continue our data analysis using the cell health parameter. Overall, these results demonstrate the ability of the DET3Ct platform to rapidly give quantitative drug sensitivity measurements.

When comparing drug response between tissue and ascites samples, no significant difference was observed between these groups (Supplementary Fig. 2f). This is the same for the four patients where both sample types were tested (Supplementary Fig. 2g). Further, examination of the four paired samples revealed that cells from ascites were more sensitive to drugs targeting both mammalian target of rapamycin (mTOR) and phosphoinositide 3-kinase (PI3K) inhibitors, as three of the drugs with the most differential response are annotated with these targets (Fig. 2b). In addition, mepacrine and close structural analogues have also been reported to exert their cytotoxic effect through this pathway^16^. Ascites fluid and spheroid aggregates have previously been shown to have high activation of mTOR and PI3K pathways, suggesting the maintenance of this niche dependence in our short-term ex vivo cultures. Overall, these data indicate that ascites could serve as an easily accessible source of cancer cells for future diagnostics.

Examining the patterns of drug sensitivity and resistance across the patient cohort revealed that none of the drugs were effective in all samples. For the standard of care drugs cisplatin and carboplatin 13 and 8 samples, respectively, were classified as sensitive to these drugs (DSS > 8, Supplementary Fig. 2h). Other drugs effective in more than half of the samples were: omipalisb, a PI3K/mTOR inhibitor, A-1331852, a Bcl-xL inhibitor, omacetaxine, a protein translation inhibitor and dactinomycin, a DNA intercalator. The dependency of OC on Bcl-xL, encoded by *BCL2L1*, has been previously described^17–19^, and is supported by our drug response data. In line with this, the Bcl-2 selective inhibitor, venetoclax, is only active in a single sample, further pointing to a reliance of OC on Bcl-xL anti-apoptotic signaling. Inspecting individual patient responses to the OC repurposing library revealed that for all patients at least one effective drug (DSS > 8) could be identified (Fig. 2c). Interestingly, the patient diagnosed with papillary carcinoma, showed a unique drug response pattern, including erdafitinib (a fibroblast growth factor receptor inhibitor), dexamethasone, (an immunomodulatory agent) and PF-00562271, (a focal adhesion kinase (FAK) inhibitor). For each individual patient, the number of effective drugs varied between 1 and 26 with a mean of 8 per patient, suggesting future opportunities for *f*PM to identify effective drugs for OC patients. The median overall response of the patients diagnosed with LGSOC were the lowest in the cohort. This is in agreement with clinical observations that response of LGSOC patients to chemotherapy is low^17^. However, a larger cohort would be required to confirm the latter finding and to look for drugs with efficacy in specific disease subtypes.

In the cohort of 16 patients, 11 patients achieved a complete response (CR), while 4 patients had partial response (PR) and one progressive disease (P) as measured by the Response Evaluation Criteria In Solid Tumors (RECIST) scale (Supplementary Table 4). Interestingly, we observed an association between the number of effective drugs identified in a patient sample ex vivo and clinical response to treatment, where samples of patients with a CR are overall more sensitive to the drugs in the OC repurposing library (Fig. 2d). However, the number of effective drugs was not significantly different for patients with a progression free interval (PFI) ≤ 12 months and PFI > 12 months (Fig. 2e). To understand the clinical applicability of the DET3Ct platform we compared the observed DSS for standard of care therapy carboplatin to the clinical response (Table 1). Carboplatin DSS did effectively discriminate between patients achieving CR and patients with PR or P (Fig. 2f). In addition, carboplatin DSS distinguishes between patients with a PFI less than or equal to 12 months and a PFI greater than 12 months (Fig. 2g). PFI is currently used to determine second line therapy in OC and is highly associated with overall survival in line with our observations^20,21^. Interestingly, ex vivo response to cisplatin does not predict clinical response but higher response to cisplatin is associated with longer PFI (Supplementary Fig. 2i, j). The same is true for adavosertib, a WEE1 inhibitor, which distinguishes between patients with PFI less than or equal to 12 months and a PFI greater than 12 months (Fig. 2h). Adavosertib has previously been shown effective in combination with carboplatin^22^ or gemcitabine^23^. To determine if the observed results are due to the heterogeneity in 3D growth morphologies between the different samples, we investigated association of volume of individual objects or sum of all cells in negative control wells with drug response scores or patient response (Supplementary Fig. 2k–q). Unlike carboplatin these metrics did not distinguish between the clinical observations nor patients with greater than 8 or less than or equal to 8 effective drugs identified using DET3Ct (8 is the average across the cohort). Overall, these results imply that the DET3Ct platform can identify clinically relevant drug sensitivity and resistance patterns in primary patient cells.

### 3D culture shows enhanced ex vivo fold growth

**Fig. 3:**
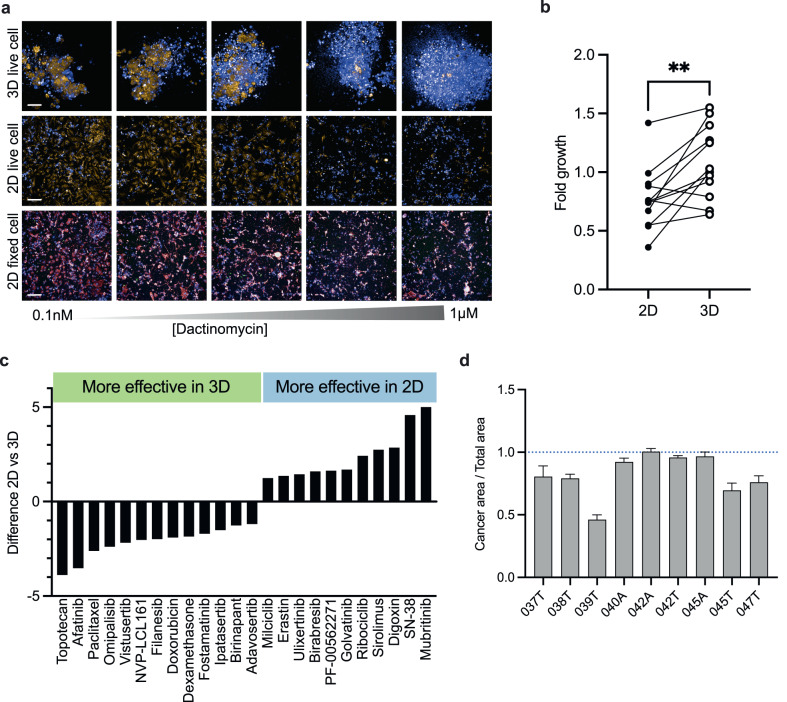
Comparison of the DECT3Ct platform results in 2D and 3D formats. **a** Example images from the patient sample OvCa037 displayed in 3D live cell assay (top panel, maximum projection), 2D live cell assay (middle panel) and 2D IF assay (bottom panel) after drug exposure to dactinomycin in a five-point concentration range. For top and middle panels TMRM is displayed in yellow, POPO-1 in blue. For the lower panel red is CK8/18 and blue is Hoechst. The scale bar for the 3D live-cell images represents 100 µm and for the 2D images is 200 µm. **b** Growth in the 2D and 3D culture conditions measured by fold change in area (2D) or volume (3D) for DMSO controls at t = 2 h and t = 72 h. Two-tailed paired *t* test, *p* = 0.0033. **c** Mean difference in drug response between 2D and 3D culture formats for the 11 paired samples. Only drugs with a DSS difference <-1 or >1 are shown. **d** Ratio of cancer area/total area in the DMSO wells for the 2D IF assay. Cancer area is determined using CK8/18 positive area (epithelial cells) and total area is determined using CellMask positive area. Error bars represent S.E.M of twelve replicates.

We evaluated drug sensitivity in 11 paired samples in both 3D and 2D assay formats to identify which one that is the most effective for our platform with regards to experimental and analytical resources needed. Assay conditions and image analysis were adapted to the 2D cultures based on the principles of the 3D assay system. Example images of paired 2D and 3D cultures are displayed in Fig. 3a, demonstrating the ability of the 2D culture and accompanying assay and analysis to capture drug response (Supplementary Fig. 3a). Notably the fold growth during the assay was significantly higher in 3D than 2D (Fig. 3b) indicating that the 3D short term cultures better retain cell proliferation. There is no significant difference in DSS between the 2D and 3D samples for individual drugs or between the two formats (Supplementary Fig. 3b). However, only 3 of 11 paired samples cluster in drug response space, suggesting that culture format also impacts drug sensitivity (Supplementary Fig. 3c).

To investigate this further we looked at the average differences in drug response between the two formats, as well as the number of hits (DSS > 8) identified in the two conditions (Fig. 3c, Supplementary Fig. 3d). For carboplatin the same patients had a DSS > 8 in both the 3D and 2D settings. Interestingly, we found that the two topoisomerase inhibitors, topotecan and SN-38, have opposite sensitivity profiles with topotecan being more effective in the 3D setting. Topoisomerase inhibitors have previously been shown to demonstrate variable efficacy in OC patient cells but not in conventional cell lines^24^. Several studies suggest that there is higher drug efficacy in 2D than in 3D models^25–27^. However, slower mitosis has been observed in 2D models of OC, which could account for enhanced activity of topotecan in the 3D models^28^. Notably, cells cultured in 2D were in general more sensitive to mubritinib. While initially described as a HER2 inhibitor, mubritinib has recently been identified as a direct, ubiquinone-dependent ETC complex I inhibitor and has been exploited to target oxidative phosphorylation (OXPHOS)-dependent leukemia cells^29^. This suggests a differential dependence on OXPHOS between the 2D and 3D culture systems, which is supported by several studies concluding that in vivo metabolism is better retained in 3D models^30–32^. In addition, afatinib, an epidermal growth factor receptor (EGFR) inhibitor, showed better sensitivity in the 3D cultures. Studies in lung cancer have found similar differences in 2D and 3D response to EGFR inhibitors, driven by increased expression of EGFR in 3D cultures^33^. Combined our data show that growth is better retained in the 3D culture setting and suggest that this culture method better models in vivo characteristics.

To determine the cellular composition of the samples we performed immunostaining (IF) on the 2D samples after live-cell drug testing, using CK8/18, as a proxy for cancer cells and FSP1 as fibroblast marker. By examining the DMSO controls of all 2D samples, we found that the majority of the cells were CK8/18 positive (Fig. 3d). A smaller fraction of cells was FSP1-positive indicating that most of the cells in the short-term cultures were of epithelial origin. The exception was OvCa039T where less than half the cells scored as epithelial origin in the 2D culture. This sample did not pass quality control in the 3D DET3Ct assay. For several samples we evaluated the drug response score in the cancer compartment only, based on CK8/18 scoring. There was a significant difference in DSS between the fixed and live-cell setting in the 2D assay format across the OC repurposing library (Supplementary Fig. 3e). Thus, in order to implement this type of assay, additional patient samples would need to be evaluated to determine predictivity and benefits of this type of analysis.

### Patient-specific combination screening reveals afatinib and A-1331852 to be an effective drug combination

**Fig. 4:**
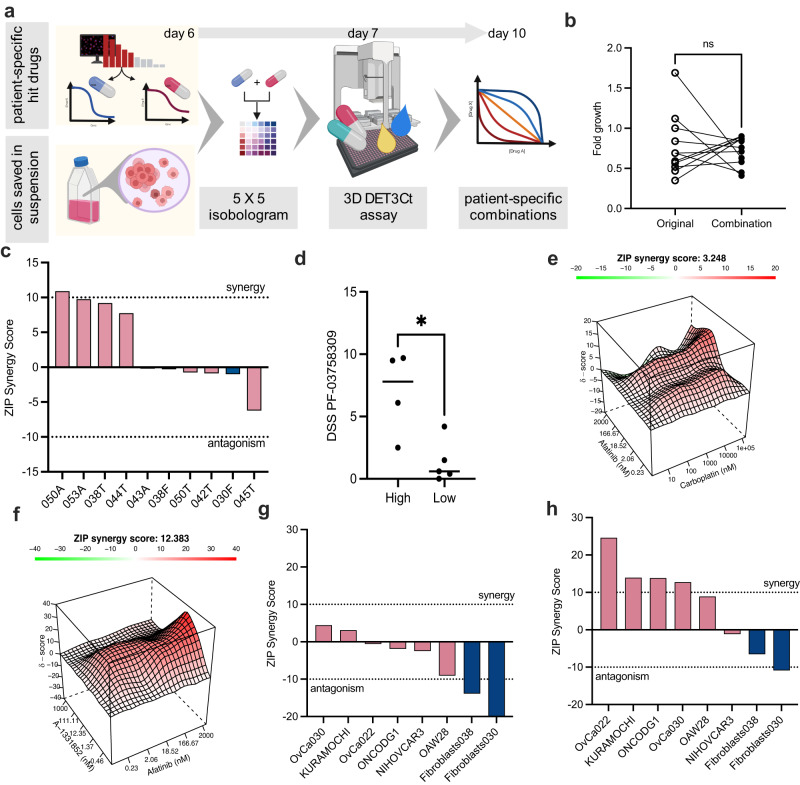
Patient specific combination screening reveals a synergistic interaction. **a** An overview of the combination testing strategy. Figure created in BioRender.com. **b** Comparison of the fold growth (TMRM volume) during the initial screen (days 3–6) and the following combination screen (days 7–10). **c** ZIP synergy scores of the combination A-1331852+carboplatin from the patient-specific combination screens using the 3D DET3Ct method for the 6 patient samples (pink, A or T) and 2 patient-derived fibroblast lines (blue, F). **d** DSS scores of PF-03758309 for the samples in c (4 with ZIP synergy scores >7 and 5 with ZIP synergy scores <0). Two-tailed Mann–Whitney *U* test *p* = 0.0317. **e** Interaction surface for the combination matrix of afatinib (five concentrations) 0.2–2000 nM and carboplatin (five concentrations) 10–100000 nM. **f** Interaction surface for the combination matrix of afatinib (five concentrations) 0.2–2000 nM and A-1331852 (five concentrations) 0.5–1000 nM. **g** ZIP synergy scores of the combination afatinib and carboplatin using the 3D DET3Ct method for 6 OC cancer models (pink) and two PDF models (blue). **h** ZIP synergy scores of the combination afatinib and A-1331852 using the 3D DET3Ct method for 6 OC cancer models (pink) and 2 PDF models (blue).

Resistance is a major challenge for single-agent, targeted therapies and methods to evaluate patient-specific combinations are of great need. The DET3Ct platform allows not only for rapid drug efficacy profiling, but also lends itself to patient-specific combination screening. When sufficient material was available, primary patient cells were maintained in suspension culture during the initial efficacy profiling. Drug combinations were then selected based on the individual drug sensitivity profile for each patient and we tested the complete matrix of 5 × 5 concentrations of each drug (Fig. 4a). Overall, we evaluated tailored combinations prospectively in nine different patient samples (*n* = 3 per patient). Since combinations of carboplatin were tested in all samples, we compared the DSS scores from the original profile and the follow up test and found no significant difference. (Supplementary Fig. 4a). In addition, no significant difference in the fold growth over the 72 h-treatment period from the samples of the initial drug profiling screen compared to the samples from the follow up combination testing screen was observed (Fig. 4b). Of the 27 examined combinations, 5 were synergistic (ZIP > 10), 22 additive (−10 < ZIP > 10) and 0 antagonistic (ZIP < −10) (Supplementary Table 6). Since A-1331852 was effective in many of the patient samples, it was tested in a high proportion of the patient-specific drug combination screens in combination with carboplatin (Fig. 4c). Interestingly, Bcl-xL expression and has previously been associated with chemoresistance and recurrent disease in OC^34^ and has been associated with chemotherapy resistance in OC cell lines^35^. In our study, this combination showed an additive effect in four samples. The positive additive effect was not observed in two patient-derived fibroblast (PDF) models, suggesting that the combination shows some selectivity for the epithelial cancer compartment. Interestingly, DSS of the PAK inhibitor (PF-03758309) distinguishes between samples with high additivity (ZIP synergy score >7) and those with low additivity (ZIP synergy score < 0), suggesting that a common marker of drug synergy could exist. Given the role of Bcl-xL in recurrent disease, and the observed high additivity of this combination in patient samples, this combination would be worth exploring in a clinical setting.

We evaluated two combinations with the EGFR inhibitor, afatinib, in a prospective patient sample, one with carboplatin and the other with A-1331852 (Fig. 4e, f, Supplementary Fig. 4b, c). Afatinib and A-1331852 combination gave synergy, thus we wanted to determine the generality of this combination. First, we selected a cell line panel with varying sensitivity to afatinib and A-1331582, (Supplementary Fig. 4d), as well as two PDC models and two PDF models. We then evaluated the combination of afatinib and carboplatin using the 3D DET3Ct assay (Fig. 4g). For this combination antagonism was seen in the fibroblast models, whereas the cancer models generally yielded moderate additivity. We observed that the OAW28 cell line had a negative ZIP score, close to antagonism. Interestingly, OAW28 was the only cell line in the panel that was sensitive to the Mcl-1 inhibitor S-63845 and displayed the highest sensitivity to the dual Bcl-2/Bcl-xL inhibitor navitoclax. Gene expression data shows that OAW28 has the highest levels of Bcl-2, encoded by *BCL2*, in the cell line panel (Supplementary Fig. 4e). This suggests that OAW28 can adapt to the inhibition of Bcl-xL through other anti-apoptotic counterparts, such as Bcl-2 and Mcl-1. Notably, high Bcl-2 levels have previously been associated with resistance to chemotherapy, such as carboplatin reviewed in^19^, as seen in OAW28. For the combination of afatinib and A-1331852, a synergistic effect could be observed in 4 out of the 6 OC models, while both the PDF models had a negative ZIP score (Fig. 4g). This indicates that this combination effect might be selective for the epithelial cancer cells. The two cell lines for which synergy was not observed were OAW28 and NIHOVCAR3. As mentioned above OAW28, has high expression of Bcl-2, which could mitigate dependence on Bcl-xL. Additionally, although the ONCO-DG-1 cell line has been identified as a derivative of NIHOVCAR3^36^, they respond differently to both afatinib and the combination of afatinib and A-1331852. Comparison of publicly available transcriptomic data shows that the two cell lines are indeed highly similar, although the NIHOVCAR3 cell line has significantly higher *SPARC* expression (Supplementary Fig. 4f). Secreted SPARC has been shown to influence growth-factor signaling in OC which could explain our observations^37^, but further investigation is necessary to confirm this finding. Thus, we demonstrate here that the DET3Ct approach can be used for identification of effective combinations in 10 days.

### A-1331852 and afatinib in combination show increased efficacy in long-term culture

**Fig. 5:**
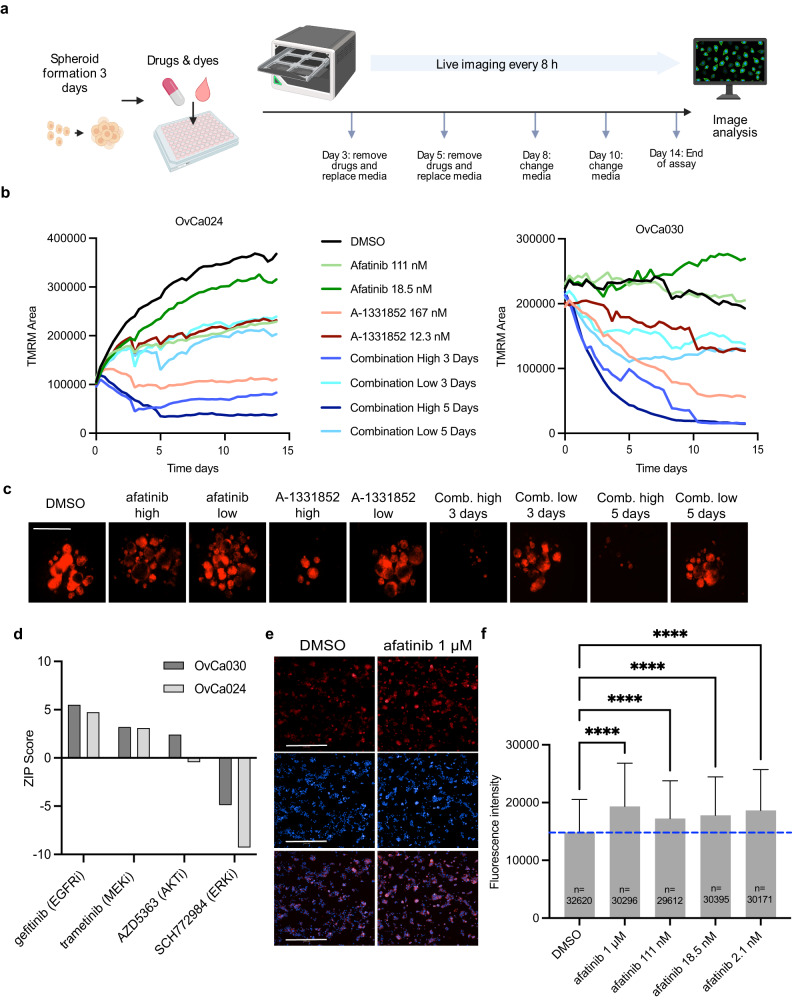
Combination of A-1331852 and afatinib has promising effects in long-term, washout model. **a** A schematic overview of the assay. **b** TMRM area in OvCa024 and OvCa030 plotted over 14 days, where the drugs afatinib and A-1331852 were used in combination and as single agents. A-1331852 (Low: 12.3 nM and High: 167 nM) and afatinib (Low: 18.5 nM and High: 111 nM). Imaging was performed every 8 h. Mean value at each timepoint is shown for ease of visualization. **c** Representative images of OvCa030 from different treatments at day 14 (red-orange is TMRM intensity). Scale bar is 400 µm. **d** Combination of A-1331852 with gefitinib, trametinib, AZD5363, and SCH772964 in OvCa030 and OvCa024 models measured with the 3D DET3Ct assay. **e** Representative images of BIM expression in DMSO control cells (left) and cells exposed to 1 µM afatinib. BIM expression is depicted in red and Hoechst (nuclei) is blue. Top panel is BIM, middle is Hoechst and bottom is merged. Scale bar is 500 µm. **f** Quantification of BIM expression after afatinib treatment at the concentrations 1 μM, 0.111 nM, 18.5 nM and 2.1 nM compared to DMSO control measured with high content microscopy. One-way ANOVA, *p* < 0.0001 for all conditions as compared to DMSO (ctrl), number of cells evaluated is shown for each condition. Barplots show median and interquartile range.

Removal of drug treatment, especially of targeted therapies, can result in re-growth of the tumor, indicating cytostatic rather than cytotoxic effects. In order to examine the pharmacokinetics of this process and compare the long-term effect of monotherapies to combination therapies, we established an assay incorporating drug washout. In short, aggregated cells were exposed to the single agents and the combination of A-1331852 and afatinib over a period of 14 days in culture (Fig. 5a). Drug treatments were renewed or washed out on days three and five and media was refreshed every three days until completion of the assay. Four PDC models, OvCa022, OvCa024, OvCa027 and OvCa030 were used for these experiments (Fig. 5b, c, Supplementary Fig. 5a, b, Supplementary Table 1). For all PDC models, the combination treatment considerably outperformed the single agents, such that almost all the cells were dead at day 10 and no re-growth observed on day 14. Afatinib alone shows little to no effect on growth rate as compared to the control in all cultures and for the lowest concentration increased cell proliferation can be observed in OvCa030 (Fig. 5c).

To investigate the underlying mechanisms of the observed synergy, we examined several inhibitors connected to the EGFR-RAS-RAF-MEK signaling pathway. With the reversible EGFR inhibitor gefitinib in combination with A-1331852, a positive synergy score was observed (Fig. 5d). This was the same for a MEK inhibitor, trametinib, and an AKT inhibitor, AZD5363, although to a lesser degree than the EGFR inhibitors. In addition, we had observed synergy for one additional prospective patient sample with a MEK inhibitor (Supplementary Table 6). Notably, with the ERK inhibitor SCH772984, a negative ZIP score was observed in both PDC models when combined with A-1331852. ERK interacts directly with pro-apoptotic factors, such as BIM (encoded by *BCL2L11)*, as well as modulates expression of its anti-apoptotic counterparts, Bcl-xL and Bcl-2, to regulate cell fate^38^. In line with these findings we also examined Bcl-xL and BIM upon treatment with afatinib and found that both Bcl-xL and BIM levels were increased upon treatment (Fig. 5e, f, Supplementary Fig. 5b). In conclusion, we hypothesize that EGFR inhibitors upregulate BIM leading to the observed synergy with the Bcl-xL inhibitor A-1331852 (Supplementary Fig. 5c) and that this has the potential to target OC cells.

## Discussion

Here, we aim to develop a robust *f*PM technology for rapid, ex vivo drug sensitivity testing to complement current precision medicine approaches based on molecular characterization. Current *f*PM approaches for solid tumors typically use organoids and PDC models. Both strategies suffer from long time frames, as it takes weeks or months for model establishment and expansion^10,11^. This is not compatible with treatment timelines in many diseases and will increase the risk of geno- and phenotypic drift of the model. Furthermore, protocols for PDC and organoid establishment are far from being 100% successful, precluding equitable and unbiased clinical implementation of *f*PM approaches in solid tumors at present.

We present a scalable drug testing platform, DET3Ct, where primary cells can be rapidly analyzed with turnaround times compatible clinical decision timelines. We also describe how patient-specific drug combinations can be subsequently selected and evaluated based on each individual patient’s drug profile with results available within ten days from sampling. The advantage with the DET3Ct platform is that it exploits fresh, uncultured cells, making it notably faster than most of the other *f*PM strategies in solid tumors. In addition, 90% of the samples passed quality control in the drug testing, removing the potential bias seen in model generation. We observed that a large proportion of the cells were of epithelial origin in our short-term cultures, whereas during 2D model generation, fibroblast outgrowth is a major challenge^39^. This further supports the use of prospective, short-term cultures for future *f*PM implementations and even the positioning of drug candidates.

We demonstrate this technology in OC, a disease where treatment is not currently tailored to individual pathobiology and there has been little improvement in patient outcome in the past 25 years^14^. We did not identify a single drug that showed efficacy across all the patient samples. This highlights the heterogeneity of the ex vivo drug response and the urgent need for personalized treatment strategies in OC. DSS for carboplatin, and cisplatin from the DET3Ct platform could successfully distinguish between patients with a PFI ≤ 12 months and those with a PFI > 12 months. Since combinations of platinum drugs are the mainstay of maintenance therapy for this patient group, and PFI is currently used to decide second-line treatments, the DET3Ct platform could be used to better stratify patients to treatment, or to deselect patients for surgery, in the future. Interestingly, adavosertib could also discriminate between patients in these two groups, which suggests its efficacy in platinum sensitive patients. Recent clinical trials in this patient group show similar results^22,23^. Notably, we observed ex vivo efficacy of on average eight drugs per patient suggesting opportunities for drug repositioning to this disease setting, and improving patient treatment strategies in the future.

While differences in 2D and 3D cultures methods are relatively well documented in OC cell lines in OC, few examples in primary cells are reported^25,35,40^. For example, a study comparing organoid drug response cells cultured in 2D showed that drug sensitivity was not generally decreased in organoids. Instead, drug responses were more diverse and correlated better with genomic alterations in the 3D culture as compared to 2D culture^41^. When we examined the paired patient samples, we found that cells cultured in 2D were in general more sensitive to mubritinib, suggesting a dependence on OXPHOS in the 2D setting. This observation, combined with the retained proliferation rate, leads us to favour the use of the 3D model for further development.

One unique aspect of our study is the use of patient-specific drug profiles to rationally design and evaluate combinations on a clinically relevant time frame. In the present study, we found a positive additive effect between carboplatin and A-1331852 in four of the patient combinations screens. Taken together, Bcl-xL inhibitors combined with current treatment regimens might be a strategy worth considering to improve outcomes for OC patients in the future. A challenge with Bcl-xL inhibitors is the reported on-target toxicity, limiting clinical applications of these drugs. Thus, there has been a major effort focused on new therapeutic modalities to target this protein^42^, as well as strategies aimed at reducing therapeutic doses. One potential method is synergistic combinations, enabling use of lower Bcl-xL doses. Several recent studies in OC have explored combinations with A-1331852 and these propose synergy with PI3K inhibitors, MEK inhibitors or an FGFR4 inhibitor^43–45^. Interestingly, while running patient specific combination screening, we found a synergistic effect when combining afatinib (EGFR/HER2 inhibitor) and A-1331852 (Bcl-xL inhibitor). Further validation of this combination was promising, since most of the cancer models displayed a synergistic effect (4 of 6), which was not observed in the PDFs (Fig. 4f). In addition, we confirmed the long-term efficacy of this combination in wash-out studies with 14 days of treatments (Fig. 5a–c). To the best of our knowledge this drug combination has not been proposed earlier in OC. Despite the promise of EGFR inhibitors in OC, multiple clinical trials have not found an application for these in patient care. In non-small cell lung cancer, on the other hand, this combination has been explored previously and one study found that combined treatment with osimertinib (EGFR inhibitor) and navitoclax (Bcl-2 and Bcl-xL inhibitor) caused synergistic inhibition on cell growth^46^, which is driven by BIM upregulation^47^.

We observed a significant increase of both Bcl-xL and BIM upon treatment with afatinib in one of our models. We hypothesize that inhibition of EGFR alone does not lead to sufficient BIM induction to cause cell death, but when introducing a second insult, *i.e*. Bcl-xL inhibition, the cells become primed for apoptosis. This is in line with the findings in the studies proposing combinations of MEK or PI3K with Bcl-xL or Bcl-2 inhibitors, which converge on similar cellular signaling. In fact, in our hands, combinations with the EGFR inhibitors outperformed the MEK and AKT inhibitors we tested. Previous results suggest that p-BIM upregulation after MEK inhibitor exposure can be used as a biomarker for patient stratification, suggesting a potential biomarker for further follow-up^43,44^. The negative synergy score observed with the ERK inhibitor SCH772984, suggests that although ERK inhibition frees additional BIM, it also impacts the expression of Bcl-xL or other counterparts as observed previously^48^. Our studies in PDFs suggest that the observed synergy is selective for the epithelial compartment. Based on these findings, our study provides a potential strategy to minimize Bcl-xL doses for OC patients through pharmacological stimulation of BIM.

In conclusion the drug profiling platform DET3Ct with a combination testing capability could in the future be useful for identifying existing and emerging drugs and drug combinations for repurposing in OC. However, further refinement and improvement to our assay such as cell-specific drug response in 3D might be needed to be able to use it in clinical settings in the future. For example, due to the miniaturization of the method we may not be able to capture cellular heterogeneity in individual patients and the short-term assay concept is not currently adapted to identify persister cells. Nonetheless, using the DET3Ct assay, we identify combination treatments of the Bcl-xL inhibitor A-1331852 with afatinib or carboplatin could potentially be of benefit for OC patients in the future.

## Methods

### Method overview

Tumor tissue, and in some cases ascites fluid, are taken directly from surgery and processed into single cells/small clusters. The cells are then plated in 384-well plates, for culture in 2D and 3D. If there are left-over cells, they are saved for culture in ultra-low attachment (ULA) flasks and used subsequently. The plates are incubated for 2–3 days following addition of drugs and subsequent live-cell imaging at 0 and 72 h. The plates are then fixed and saved for immunostaining with cancer-specific and fibroblast-specific antibodies. The live-cell imaging data are analyzed for hits and the cells saved in culture are plated in 3D for follow-up patient specific combination screening, using imaging at 0 and 72 h.

### Patient samples

Patient samples were obtained from Karolinska University Hospital in Stockholm (Ethical permits from Etikprövningsmyndigheten (Swedish Ethical Review Authority): 2020-025830, 2018/2642-32, 2016/1197-31/1, 2016/2060-32). All patients signed an informed consent prior to inclusion. Sample information is presented in Table 1.

### Sample processing

#### Tissue dissociation

Tissue pieces acquired at time of surgery were processed immediately upon arrival. Several 1 × 1 mm tissue pieces were flash frozen and stored for sequencing. Remaining tissue was washed in Ultra Saline A (Lonza), minced to 2–4 mm pieces and processed using a gentleMACS tissue dissociator with manufacturer recommended protocols and reagent kits. Cells were filtered through a 70 µM strainer and centrifuged (500 g for 5 min at RT). Red blood cells were removed using ACK Lysis Buffer (Gibco) followed by washing with RPMI (Merck) containing 10% FBS (Invitrogen).

#### Ascites samples

Cells from ascites fluid were isolated by centrifugation (800 g for 10 min at 4 °C). Red blood cells were removed using ACK Lysis Buffer followed by washing with RPMI containing 10% FBS. Single cells/small clusters were plated in RockiT-medium: 3:1 (v/v) F-12 Nutrient Mixture (Ham) - DMEM (Invitrogen), 5% FBS, 1% PenStrep (Merck), 1% L-glutamine (Merck), 0.4 µg/mL hydrocortisone (Merck), 5 µg/mL insulin (Merck), 8.4 ng/mL cholera toxin (Merck), 10 ng/mL EGF (Peprotech), 24 µg/mL adenine (Merck) supplemented with 10 µM ROCK inhibitor (Y-27632, Enzo Life Sciences) and 7.5 µg/mL transferrin (Merck).

### Cell culture and drug treatment

After isolation cells were plated immediately in RockiT medium into U-bottom ULA 384-well plates (Corning) for 3D culture and Cell Carrier Ultra flat bottom 384-well plates (Perkin Elmer) for 2D culture. Remaining cells were cultured in ULA T-75 flasks in RockiT in a humidified incubator 37 °C, 5% CO_2_ with media exchange every 3–4 days until primary screen was run. Assay plates were incubated for 2-3 days and treated with 58 different drugs in 5 concentrations. Drug plates (Greiner) were pre-spotted using an Echo 550 acoustic dispensing system (Beckman Coulter). Cisplatin and Carboplatin were freshly prepared in MilliQ water and manually pipetted into the drug plates due to short expiration times. RockiT containing imaging dyes TMRM (Invitrogen), POPO-1 (Invitrogen) and Caspase 3/7 Green (Invitrogen) at final concentrations of 75 nM, 25 nM and 117 nM respectively, were added to the drug plates and lifted into the plates containing the cells with a Bravo liquid handling robot (Agilent). The cells were imaged live using an Opera Phenix (Perkin Elmer) using a stack with 12–15 planes and a 20X objective, at 0 h and 72 h post drug treatment. One hour before the endpoint imaging (72 h) of the plates 10 μg/ml final concentration of Hoechst33342 (Merck) was added to the cells.

In house developed PDC models OvCa022, OvCa024, OvCa027 and OvCa030 were maintained in RockiT. Patient-derived fibroblast (PDF) models OvCa030F and OvCa038F were maintained in 1:1 (v/v) F-12 Nutrient Mixture (Ham) - DMEM (Gibco), 10% FBS, 1% PenStrep, 1% L-glutamine, 1% Sodium pyruvate (Gibco), 1% Sodium bicarbonate (Gibco), 0.4 µg/mL hydrocortisone, 5 µg/mL insulin, 5 ng/mL bFGF (Peprotech). The cell lines ONCODG1 and KURAMOCHI were maintained in RPMI (10% FBS, 1% PenStrep, 1% L-glutamine). OAW28 was maintained in DMEM (Gibco) (10% FBS, 1% PenStrep, 1% L-glutamine, 5 µg/ml insulin (Merck)). NIHOVCAR3 was maintained in RPMI (20% FBS, 1% PEST, 1% L-glutamine, 0.1 mg/ml insulin). Commercially available cell lines were confirmed by short-tandem repeat sequencing and were mycoplasma tested. All the cell lines and models were kept in a humidified incubator 37 °C, 5% CO_2_ and split twice a week. For the combination screening the cells were seeded in U-bottom ULA 384-well plates and incubated for 3 days for spheroid formation prior to addition of drugs according to protocol above.

### Immunostaining

After imaging, the cell plates were fixed in 4% paraformaldehyde (Merck) for 20 min for monolayer cultures and 45 min for 3D cultures. The plates were permeabilized with 0.3% Triton X-100 (Fisher Scientific) for 10 min for monolayer cultures and 45 min for 3D cultures following blocking with 3% BSA (VWR) for 1 h at RT. Plates were incubated overnight at 4 °C with antibodies CK8/18 cocktail (Agilent/Dako M3652) in dilution 1:500, FSP1/S100A4 (Merck, AMAB90598) in dilution 1:400, and Bim (Abcam, ab32158) 1:100, for the validation studies in 1% BSA in PBS (0.1% Tween20). The plates were stained with secondary antibodies goat anti-rabbit Alexa Fluor 647 (Invitrogen A21244) and goat-anti mouse Alexa Fluor 568 (Invitrogen, A11004) in dilutions 1:2000 for 1 h, at RT. Hoechst 33342 (5 µg/mL in final concentration) was added to the plates and incubated for 10 min at RT. The plates were washed three times with PBS between each step using an EL406 (BioTek) liquid handling system.

### Combination screening

After analysis of the results from the initial drug screening, patient-specific drug combinations were chosen and the drugs were spotted into plates as described above. Cells from each patient were saved in 3D culture in ULA flasks in suspension and subsequently seeded in ULA U-bottom 384-well plates and used for the combination screening with the same method as described above. The combinations picked for each sample can be seen in Supplementary Table S6. The drug combination responses were calculated based on the ZIP reference model using the SynergyFinder online tool v3 with technical replicates grouped and background correction on^49^.

### Long-term combination testing and wash out studies

The PDC models OvCa022 and OvCa030 were seeded into ULA U-bottom 96-well spheroid plates (Perkin Elmer) and left for 3 days for spheroid formation. Cells were treated with combinations of A-1331852 and afatinib at different doses. For OvCa024 and OvCa030, A-1331852 at 12.3 nM (low) and 167 nM (high) was used along with afatinib at 18.5 nM (low) and 111 nM (high). For OvCa022 and OvCa027, A-1331852 concentrations of 1.4 nM (low) and 12.3 nM (high) were used, while afatinib concentration was 2.1 nM (low) and 18.5 nM (high). TMRM (final concentration 75 nM) was mixed in the media with the drugs and then the solution was added to the cells. The plates were placed into an IncuCyte S3 (Sartorius) and imaged every 8 h for 14 days. The drugs were washed out after 3 or 5 days and the media was then subsequently changed every 3 days. The images were analyzed with the IncuCyte software using the metric TMRM area.

### Image analysis

The images from the Opera Phenix were analyzed using the Harmony software (Perkin Elmer). Image analysis data was exported and transformed through an in-house developed R-script, which is available upon request. Quality control and drug response curves were further generated using Breeze software (FIMM)^50^. The synergy scores for combination screens were calculated using the SynergyFinder online tool^49^. Final output for the TMRM parameter, was generated by dividing TMRM volume by the composite spheroid volume. The composite spheroid volume was calculated the summation of intensity for all fluorescence channels and the inverted Brightfield intensity. To determine the POPO-1 parameter, we calculated the ratio of the sum of POPO-1 area to the sum of Hoechst33342 area. The variability of the POPO-1 parameter was observed to be influenced by dead cell migration towards the edge of the wells and, in those cases, positive control was set to 0.95 if the value was under 0.1.

### Reporting summary

Further information on research design is available in the Nature Research Reporting Summary linked to this article.

### Supplementary information

REPORTING SUMMARY

Supplementary information

## Data Availability

Dose reponse data and clinical information is available in Supplementary Files. Any additional requests can be made directly to the corresponding author.
