# Supplementary material for: The drug efficacy testing in 3D cultures platform identifies effective drugs for ovarian cancer patients
Source: NPJ Precis Oncol. 2023 Oct 31;7:111. doi: 10.1038/s41698-023-00463-z (PMC10618545; doi:10.1038/s41698-023-00463-z)
Supplement: Supplementary file 1 — REPORTING SUMMARY [file 41698_2023_463_MOESM1_ESM.pdf]

nature portfolio

Corresponding author(s): Brinton Seashore-Ludlow

Last updated by author(s): Jul 4, 2023

Reporting Summary

Nature Portfolio wishes to improve the reproducibility of the work that we publish. This form provides structure for consistency and transparency in reporting. For further information on Nature Portfolio policies, see our Editorial Policies and the Editorial Policy Checklist.

Statistics

For all statistical analyses, confirm that the following items are present in the figure legend, table legend, main text, or Methods section.

n/a	Confirmed	
☐	☒	The exact sample size (*n*) for each experimental group/condition, given as a discrete number and unit of measurement
☐	☒	A statement on whether measurements were taken from distinct samples or whether the same sample was measured repeatedly
☐	☒	The statistical test(s) used AND whether they are one- or two-sided<br>*Only common tests should be described solely by name; describe more complex techniques in the Methods section.*
☐	☒	A description of all covariates tested
☒	☐	A description of any assumptions or corrections, such as tests of normality and adjustment for multiple comparisons
☒	☐	A full description of the statistical parameters including central tendency (e.g. means) or other basic estimates (e.g. regression coefficient) AND variation (e.g. standard deviation) or associated estimates of uncertainty (e.g. confidence intervals)
☐	☒	For null hypothesis testing, the test statistic (e.g. *F*, *t*, *r*) with confidence intervals, effect sizes, degrees of freedom and *P* value noted<br>*Give P values as exact values whenever suitable.*
☒	☐	For Bayesian analysis, information on the choice of priors and Markov chain Monte Carlo settings
☒	☐	For hierarchical and complex designs, identification of the appropriate level for tests and full reporting of outcomes
☒	☐	Estimates of effect sizes (e.g. Cohen's *d*, Pearson's *r*), indicating how they were calculated

Our web collection on statistics for biologists contains articles on many of the points above.

Software and code

Policy information about availability of computer code

Data collection	not relelvant
Data analysis	R code was used to merge drug testing results to the drug layout information. This is documented in the methods section. All other software to produce dose-response curves that was used is available (FIMM BREEZE). Use of this is described in the methods section.

For manuscripts utilizing custom algorithms or software that are central to the research but not yet described in published literature, software must be made available to editors and reviewers. We strongly encourage code deposition in a community repository (e.g. GitHub). See the Nature Portfolio guidelines for submitting code & software for further information.

Data

Policy information about availability of data

All manuscripts must include a data availability statement. This statement should provide the following information, where applicable:

- Accession codes, unique identifiers, or web links for publicly available datasets
- A description of any restrictions on data availability
- For clinical datasets or third party data, please ensure that the statement adheres to our policy

All data is available in the supplementary tables.

Research involving human participants, their data, or biological material

Policy information about studies with human participants or human data. See also policy information about sex, gender (identity/presentation), and sexual orientation and race, ethnicity and racism.

Reporting on sex and gender	This study uses samples from patients diagnosed with ovarian cancer. Due to the nature of the disease, all patients are female.
Reporting on race, ethnicity, or other socially relevant groupings	None of these factors are reported in our study.
Population characteristics	This study takes consecutive consenting patients diagnosed with ovarian cancer.
Recruitment	Patients are asked to join the study prior to surgery and if they consent they are included in the study. Patients may withdraw from the study at all times.
Ethics oversight	Etikprövningsmyndighet (Swedish Ethical Reviw Authority)

Note that full information on the approval of the study protocol must also be provided in the manuscript.

Field-specific reporting

Please select the one below that is the best fit for your research. If you are not sure, read the appropriate sections before making your selection.

☒ Life sciences ☐ Behavioural & social sciences ☐ Ecological, evolutionary & environmental sciences

For a reference copy of the document with all sections, see nature.com/documents/nr-reporting-summary-flat.pdf

Life sciences study design

All studies must disclose on these points even when the disclosure is negative.

Sample size	In this study 20 samples from 16 individuals are included. This is a relatively small number, but is in alignment with similar studies in this area (e.g. Snjider, et al., Lancet Hematology 2017, or Chen X et. al., PNAS 2023).
Data exclusions	none
Replication	All studies were repeated and data is documented in the manuscript. When interesting or novel hits were found in the prospective screening we have validated the findings in additional cell lines or with mechanistic studies to ensure reproducibility.
Randomization	All samples were obtained in a prospective sample collection. At the time of drug testing and analysis information on clinical response to treatment and progression free interval were not known.
Blinding	as described above in randomization, we were blind to the clinical results at time of drug testing.

Reporting for specific materials, systems and methods

We require information from authors about some types of materials, experimental systems and methods used in many studies. Here, indicate whether each material, system or method listed is relevant to your study. If you are not sure if a list item applies to your research, read the appropriate section before selecting a response.

Materials & experimental systems

n/a	Involved in the study	
☐	☒	Antibodies
☐	☒	Eukaryotic cell lines
☒	☐	Palaeontology and archaeology
☒	☐	Animals and other organisms
☒	☐	Clinical data
☒	☐	Dual use research of concern
☒	☐	Plants

Methods

n/a	Involved in the study	
☒	☐	ChIP-seq
☒	☐	Flow cytometry
☒	☐	MRI-based neuroimaging

Antibodies

Antibodies used	CK8/18 cocktail (Agilent/Dako M3652), FSP1/S100A4 (Merck, AMAB90598) and Bim (Abcam, ab32158)
Validation	CK8/18- CK8/18 is a cocktail of two monoclonal antibodies. In Western blotting of A431 cell lysates, anti-CK8, clone EP17 recognizes a major band of 52 kDa corresponding to the expected molecular weight of CK8; and anti-CK18, clone EP30 recognizes a major band of 45 kDa corresponding to the expected molecular weight of CK18.<br>FSP1 - https://www.proteinatlas.org/ENSG00000196154-S100A4/subcellular<br>BIM- KO validated

Eukaryotic cell lines

Policy information about cell lines and Sex and Gender in Research

Cell line source(s)	Kuramochi (JRCB), OAW28 (Sigma Aldrich), NIHOVCAR3 (ATCC), ONCODG1 (DSMZ)
Authentication	All commercially available cell lines were sent for STR prior to the project start and are regularly checked for confirmation.<br>Patient-derived cells were validated by comparison of panel sequencing between original tissue and cell model.
Mycoplasma contamination	Patient-derived cells were not tested for mycoplasma contamination
Commonly misidentified lines<br>(See ICLAC register)	No cell lines from ICLAC list are included
