# Supplementary material for: The drug efficacy testing in 3D cultures platform identifies effective drugs for ovarian cancer patients
Source: NPJ Precis Oncol. 2023 Oct 31;7:111. doi: 10.1038/s41698-023-00463-z (PMC10618545; doi:10.1038/s41698-023-00463-z)
Supplement: Supplementary file 2 — Supplementary information [file 41698_2023_463_MOESM2_ESM.pdf]

Supplementary Information and Tables for:

The Drug Efficacy Testing in 3D Cultures (DET3Ct) platform enables identification of effective drugs and drug combinations for ovarian cancer patients

Emma Åkerlund[1], Greta Gudoityte[1], Elisabeth Moussaud-Lamodière [1], Olina Lind[1], Henri Colyn Bwanika[2], Kaisa Lehti[3,4], Sahar Salehi[4,5,6], Joseph Carlson[2,7], Emelie Wallin[2,5], Josefin Fernebro[5,6], Päivi Östling[1#], Olli Kallioniemi[1,8,#], Ulrika Joneborg[4,#], Brinton Seashore-Ludlow[1,*]

[1] Department of Oncology-Pathology, Science for Life Laboratory, Karolinska Institute, Stockholm, Sweden

[2] Department of Oncology-Pathology, Karolinska Institute, Stockholm, Sweden

[3] Department of Biomedical Laboratory Science, Norwegian University of Science and Technology NTNU, Trondheim, Norway

[4] Department of Microbiology, Tumor and Cell Biology, Karolinska Institutet, Stockholm, Sweden

[5] Department of Pelvic Cancer, Theme Cancer, Karolinska University Hospital, Stockholm Sweden

[6] Department of Women's and Children's Health, Division of Obstetrics and Gynecology, Karolinska Institutet, Stockholm, Sweden.

[7] Department of Pathology and Laboratory Medicine, Keck School of Medicine, University of Southern California, Los Angeles, CA 90089 USA

[8] Institute for Molecular Medicine Finland, University of Helsinki, Helsinki, Finland

# equal contribution

* Corresponding author, contact: brinton.seashore-ludlow@ki.se

Table of Contents

Supplementary Figure 1 3

Additional results from DET3Ct in patient-derived models

Supplementary Figure 2 4

DET3Ct in 3D primary models

Supplementary Figure 3 7

Evaluating the 2D and 3D assay concepts

Supplementary Figure 4 9

Real-time combination testing for personalized treatment identification

Supplementary Figure 5 11

Interaction between EGFR and Bcl-xL inhibitors

Supplementary Table 1 12

Clinical information for patient-derived models used in this study

Supplementary Table 2 13

Drugs included in the OC Repurposing Library

Supplementary Table 3 15

Drug sensivity scores for the two PDC models

Supplementary Table 4 16

Clinical information for the prospective samples used in the study

Supplementary Table 5 17

Drug sensitivity scores for the prospective samples

Supplementary Table 6 18

Combinations evaluated and ZIP synergy scores

Supplementary Figure 1

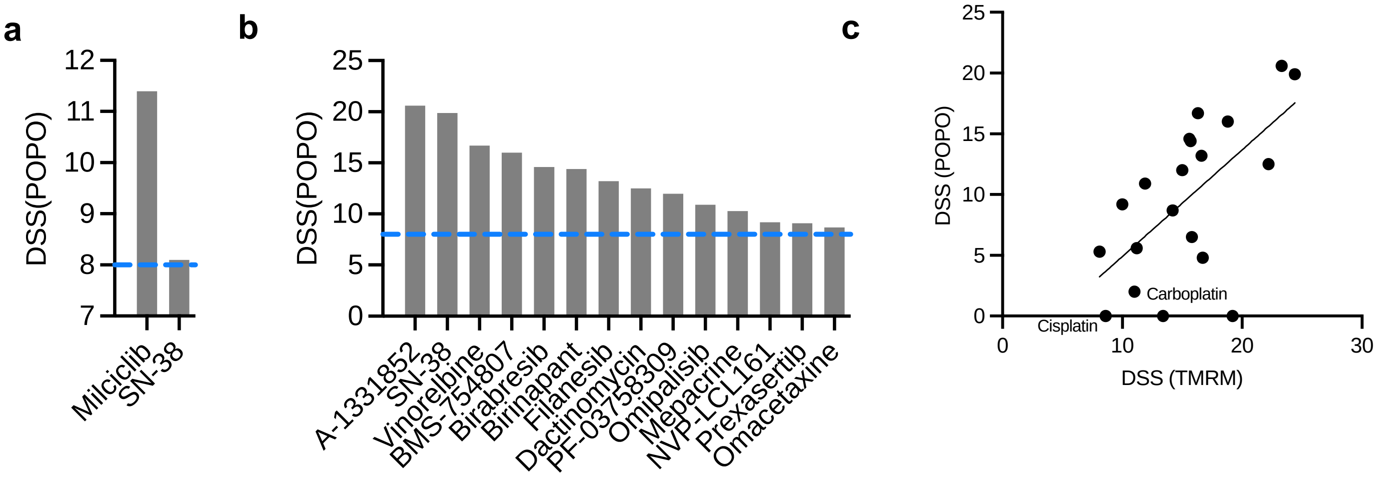

Supplmentary Figure 1. Additional results from DET3Ct in patient-derived models **a** Waterfall plot of the DSS calculated using the POPO-1 parameter for effective drugs (DSS >8) in the OvCa027 PDCs after 72-hour treatment with the OC repurposing library. **b** Waterfall plot of the DSS calculated using the POPO-1 parameter for effective drugs (DSS >8) in the OvCa024 PDCs after 72-hour treatment with the OC repurposing library. **c** correlation of DSS values from TMRM and POPO-1 measurements for the OvCa024 PDCs. Notably, carboplatin and cisplatin response differ greatly. The trend line is a simple linear regression model of the data, r^2=0.3897.

Supplementary Figure 2

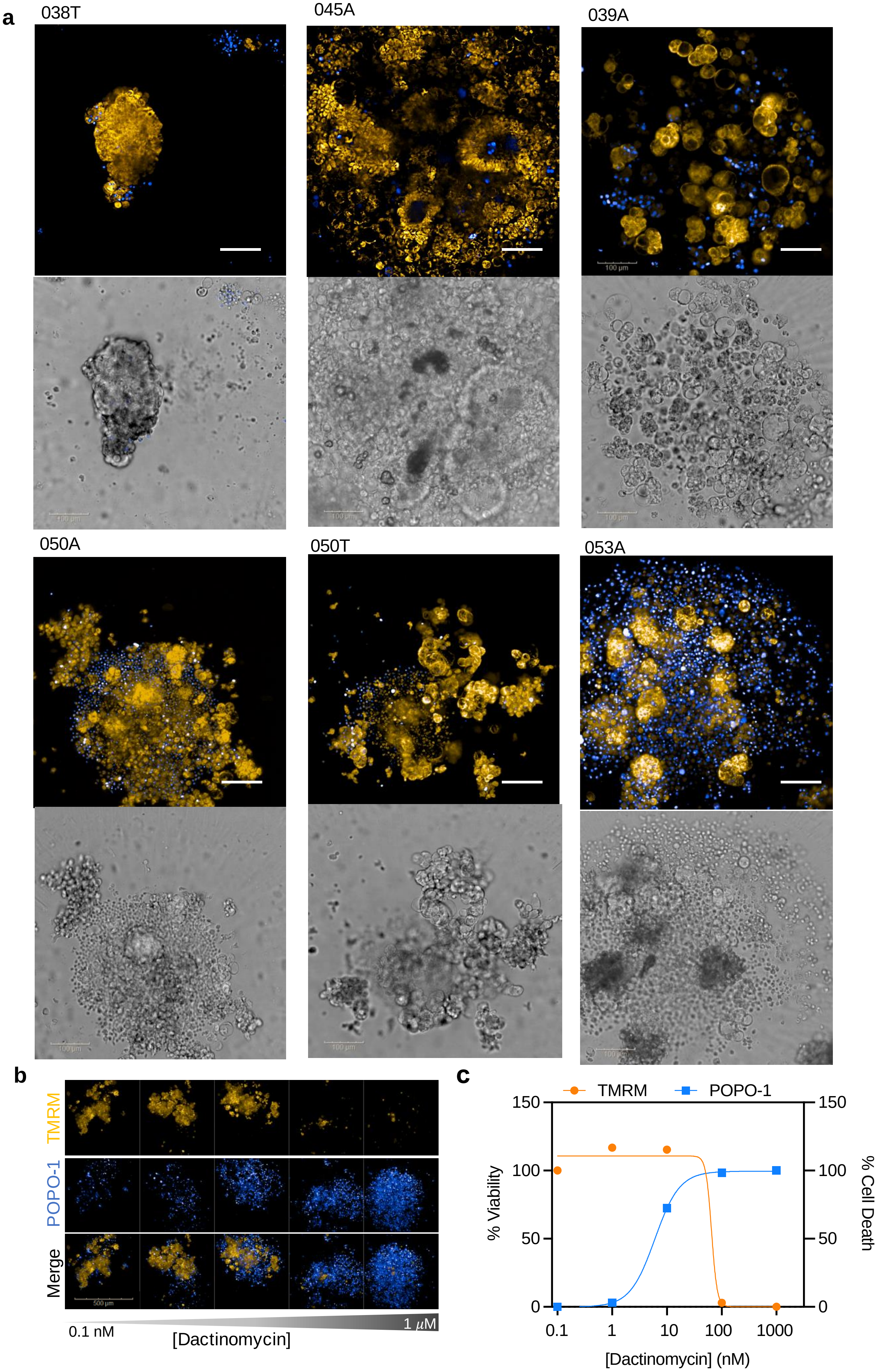

Supplementary Figure 2 continued

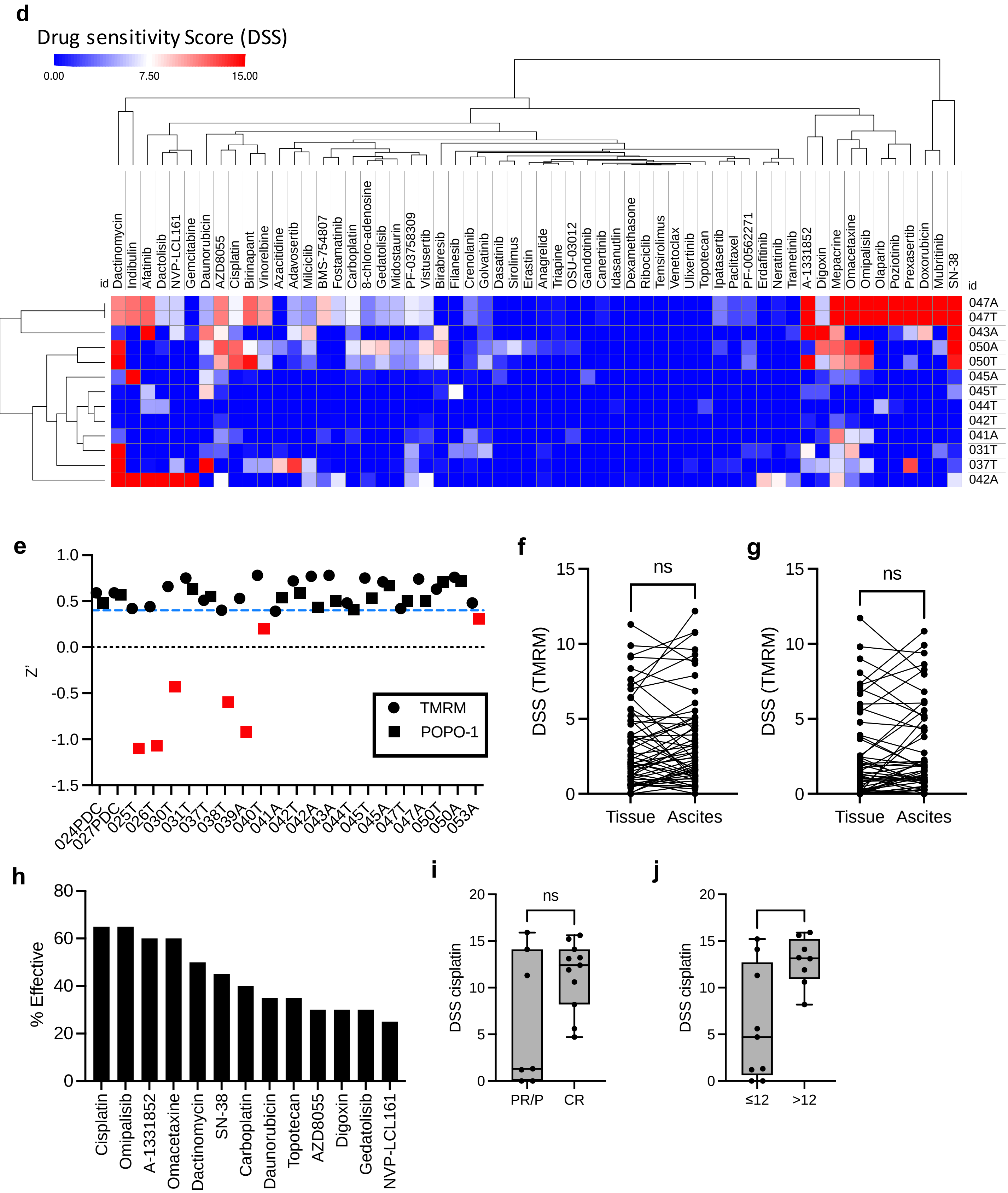

Supplementary Figure 2 continued

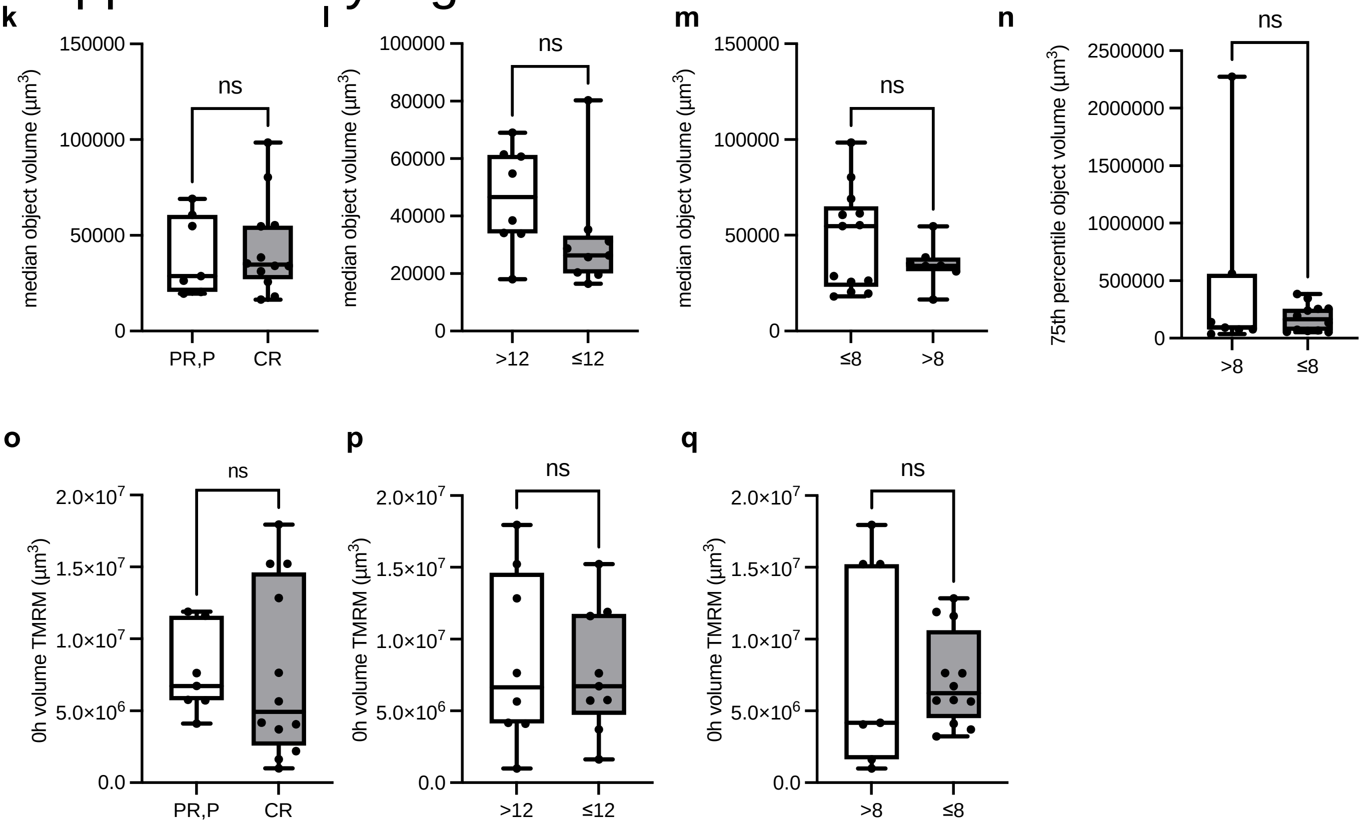

Supplementary Figure 2: Results in primary material**. a** Representative images from six patient samples, demonstrating the variation in morphology. Several samples formed single spheroids, while others formed many multicellular aggregates or cyst-like structures. For each patient shown the top panel is TMRM (orange) and POPO-1 (blue), while the bottom panel is brightfield. White scalebar represents 100 µm. **b** Representative images of the TMRM (orange), POPO-1 (blue) for five concentrations of dactinomycin. **c** Concentration response curves derived from the images in b. Cell viability is measured using TMRM (orange) and cell death is measured using POPO-1 (blue). **d** An overview of the drug response landscape presented in a heatmap showing the DSS scores from the POPO-1 parameter. Patient samples are listed on the x-axis where A stands for sample coming from ascites and T for tissue. Drugs are on the y-axis. Clustering was performed in Morpheus using Euclidean distance and complete linkage. **e** Z' values of the samples assayed in this paper. Those that above 0.4 are included in further analysis (black). **f** Average DSS for each drug in the tissue or ascites samples. Two-tailed paired t test p = 0.5052. **g** Average DSS for each drug in the tissue or ascites samples for the four paired patient samples. Two-tailed paired t test p = 0.2289. **h** The topmost effective drugs across the patient cohort calculated by number of times a drug had a DSS > 8 divided by the total number of samples in the cohort (20). **i** Boxplot of the cisplatin DSS for patients with PR/P (n=5) and CR (n =11) RECIST classes. Two-tailed Mann-Whitney U test p = 0.1868. **j** Boxrplot of the carboplatin DSS for patients with PFI ≤ 12 months (n = 7) and PFI > 12 months (n= 7). Two-tailed Mann-Whitney U test p = 0.0292. **k** Boxplot of median object volume in DMSO wells for patients with PR/P (n=5) and CR (n =11) RECIST classes. Two-tailed Mann-Whitney U test p = 0.8369. **l** Boxplot of median object volume in DMSO wells for patients with PFI ≤ 12 months (n = 7) and PFI > 12 months (n= 7). Two-tailed Mann-Whitney U test p = 0.5414. **m** Boxplot of median object volume in DMSO wells for patients with >8 or ≤ 8 effective drugs identified. Two-tailed Mann-Whitney U test p = 0.4854. **n** Boxplot of 75 percentile object volume in DMSO wells for patients with >8 or ≤ 8 effective drugs identified. Two-tailed Mann-Whitney U test p = 0.8369. **o** Boxplot of total cell volume in DMSO wells at 0h for patients with PR/P (n=5) and CR (n =11) RECIST classes. Two-tailed Mann-Whitney U test p = 0.5766. **p** Boxplot of total cell volume in DMSO wells at 0h for patients with PFI ≤ 12 months (n = 7) and PFI > 12 months (n= 7). Two-tailed Mann-Whitney U test p = 09104. **q** Boxplot of total cell volume in DMSO wells at 0h for patients with >8 or ≤ 8 effective drugs identified. Two-tailed Mann-Whitney U test p = 0.9492. Boxplots show min and max and all points.

Supplementary Figure 3

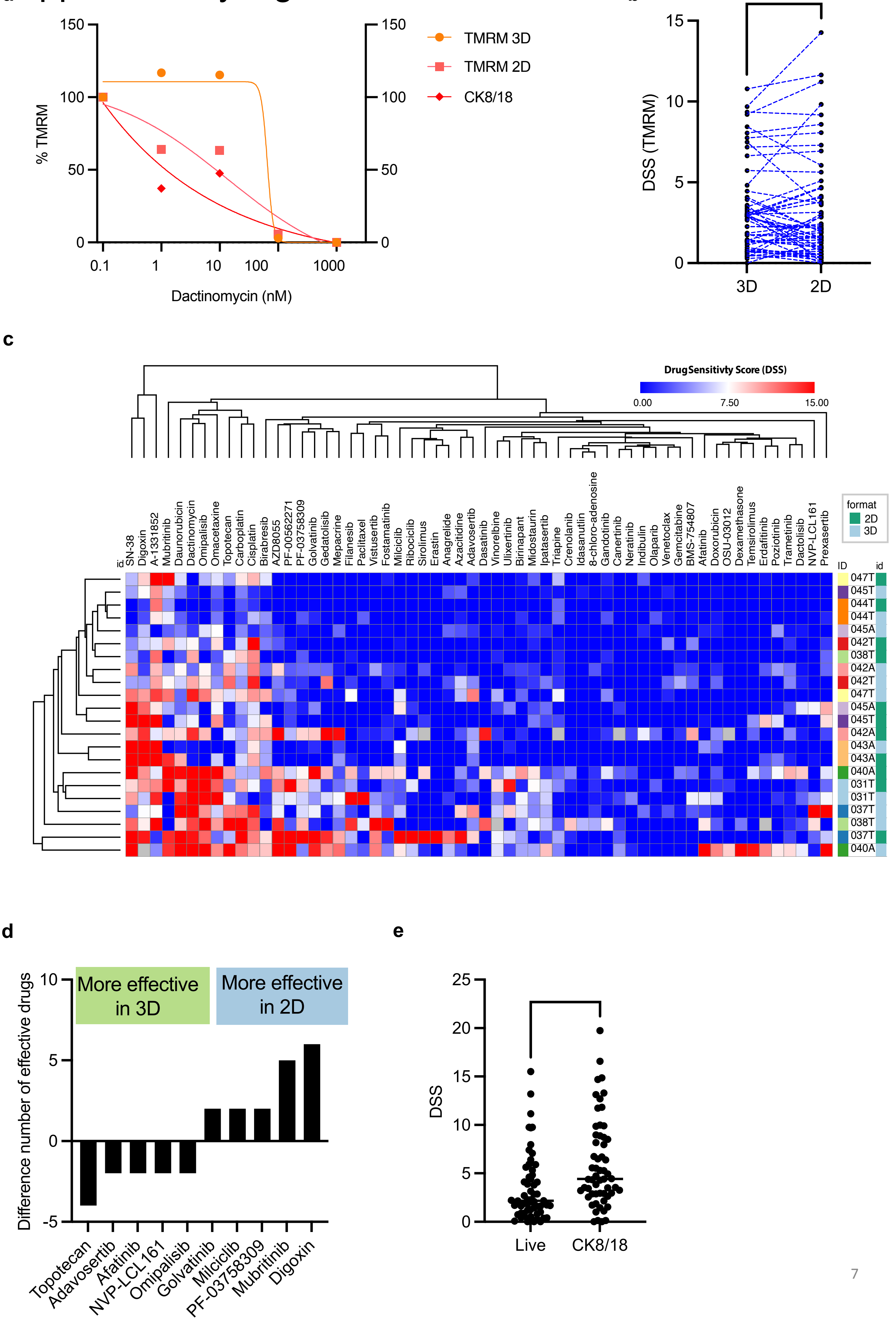

Supplementary Figure 3 cont.

Supplementary Figure S3: Additional data comparing the 2D and 3D assay**. a** Dose response curves quantified from the images in Figure 3a for 2D live and fixed cell assays from a single sample. Blue represents POPO-1 (cell death) and orange represents TMRM (cell viability). **b** Average DSS score for each compound in the OC repurposing library in the 2D and 3D samples (n = 11). There is no significant difference between the averages for the 2D and 3D settings for all drugs or for each individual drug using paired t-tests. **c** Heatmap of the 2D and 3D DSS scores for each sample-drug pair. Patient ID and assay format are found on the left (rows) and drugs are found on the top (columns). Clustering was performed in Morpheus using Euclidean distance and complete linkage. **d** Difference in the number of effective drugs identified in the 2D and 3D formats. Only drugs with a difference less than -2 or greater than 2 are shown. **e** Average DSS score for each compound in the OC repurposing library in the 2D live-cell and 2D fixed assay with quantification based on CK8/18 for 8 paired samples. Paired two-tailed t test, $p < 0.0001$.

Supplementary Figure 4

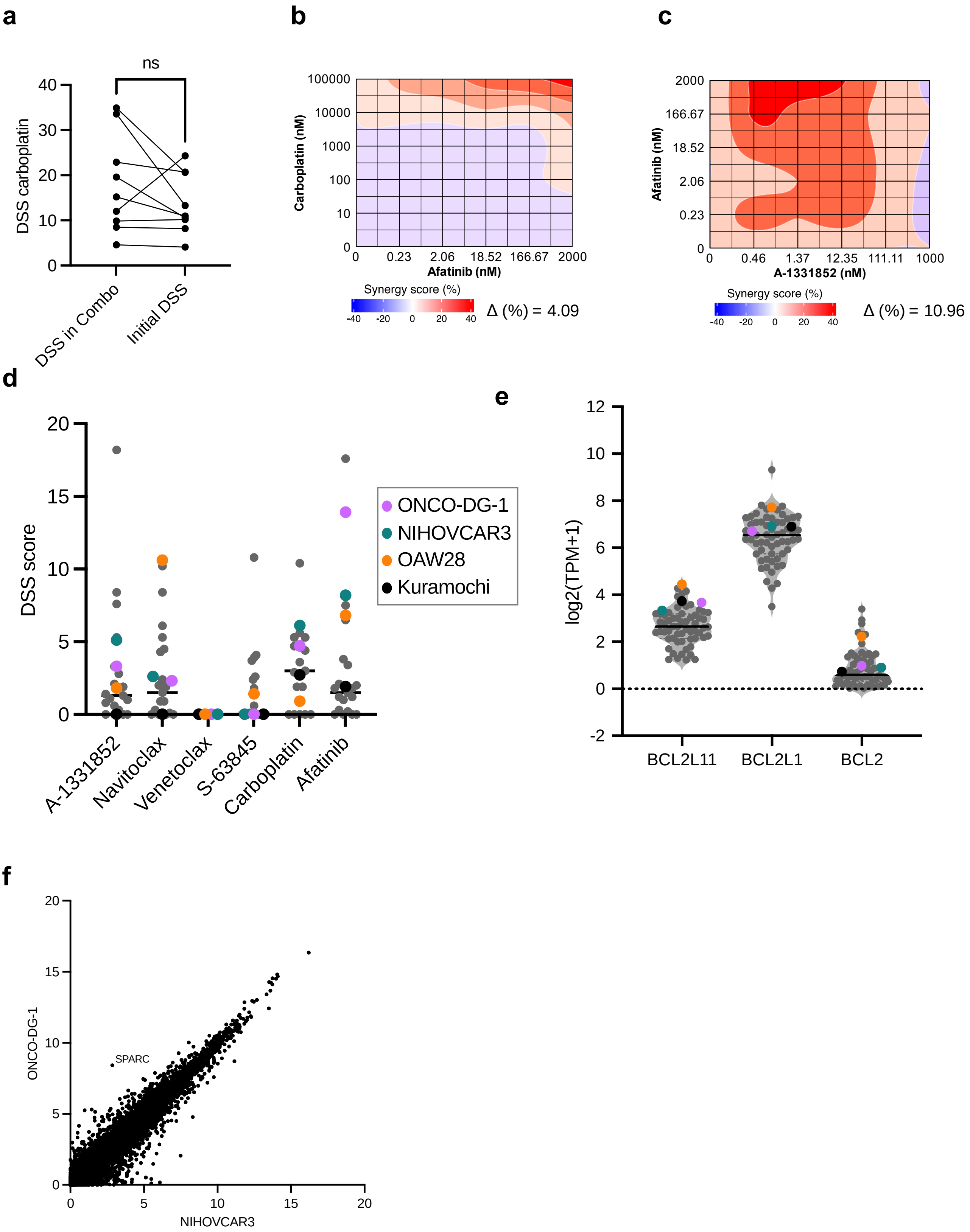

Supplementary Figure 4 cont.

Supplementary Figure 4: Real-time combination testing for personalized treatment identification**.** **a** Comparison of the DSS for carboplatin from the initial screen (days 3-6) and in the following combination screen (days 7-10). No significant difference was found with a paired Wilcoxon test p = 0.1065. **b** 2D ZIP surface plots for the combination of afatinib and carboplatin in patient sample 043A. **c** 2D ZIP surface plots for the combination of afatinib and A-1331852 in patient sample 043A. **d** DSS scores for 23 common ovarian cancer cell lines (grey dots) for selected drugs: A-1331852 (Bcl-xL), navitoclax (Bcl-xL/Bcl-2), venetoclax (Bcl-2), S-63845 (Mcl-1), carboplatin and afatinib (EGFR). The DSS for the four cell lines used in our panel are highlighted for each drug (ONCO-DG-1 (purple), NIHOVCAR3 (teal), OAW28 (orange), Kuramochi (black)). **e** Expression levels of *BCL2L11* encoding BIM, *BCL2L1* encoding Bcl-xL and *BCL2* encoding Bcl-2 from all cell lines available from CCLE with the ovarian cancer Primary Disease annotation (grey). The expression levels for the four cell lines used in our panel are highlighted for each transcript (ONCO-DG-1 (purple), NIHOVCAR3 (teal), OAW28 (orange), Kuramochi (black)). Data downloaded from Depmap portal (22Q4 dataset). Y axis represents log2(TPM+1). f Correlation of expression data for NIHOVCAR3 (x-axis) and ONCO-DG-1 (y-axis) in log2(TPM+1). Data downloaded from Depmap portal (22Q4 dataset).

Supplementary Figure 5

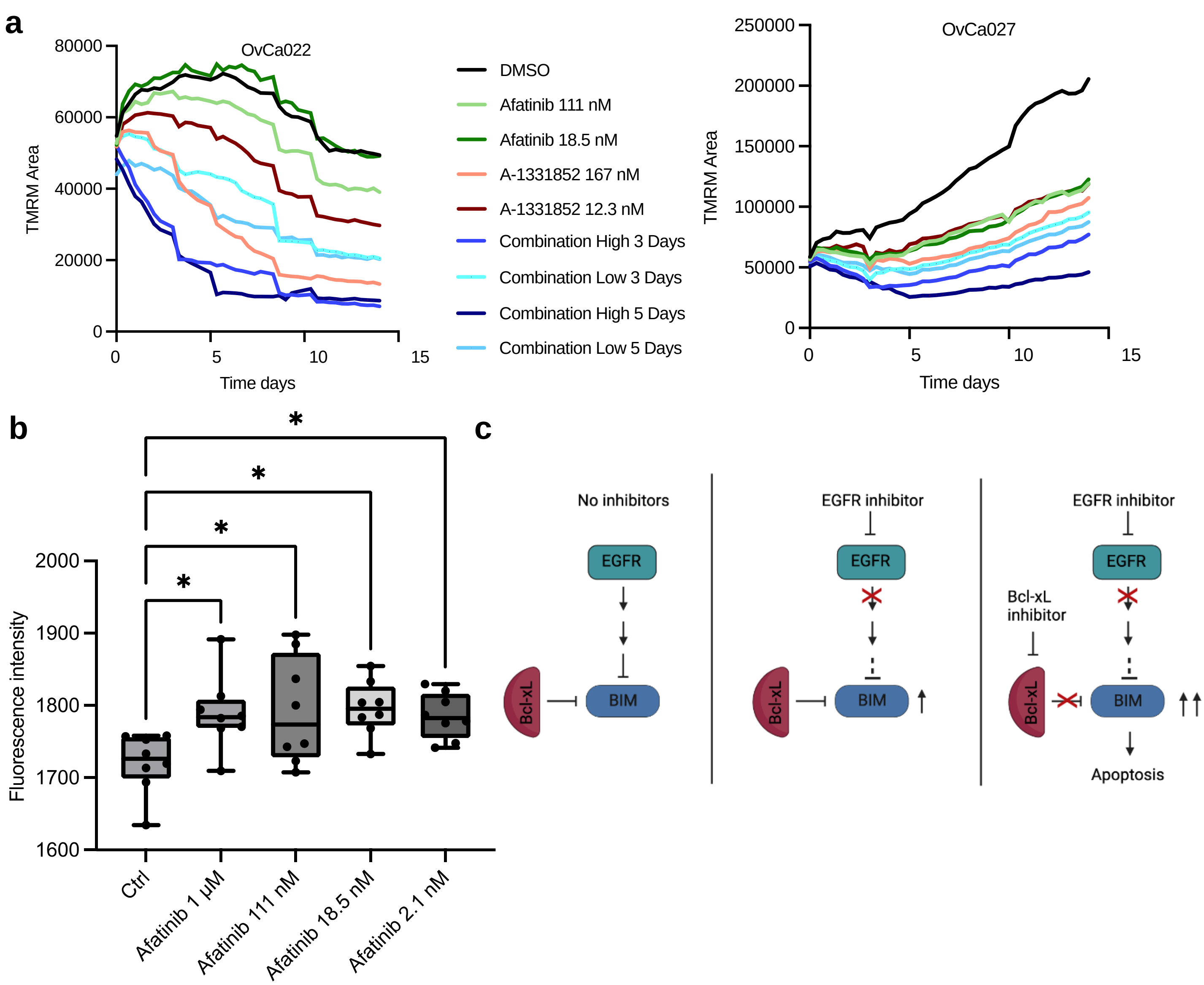

Supplementary Figure 5 Interaction of EGFR and Bcl-xL inhibitors. **a** Timeline curves of TMRM area in OvCa022 and OvCa027 over 14 days, where the drugs Afatinib and A-1331852 were used in combination and as single agents. A-1331852 (Low: 1.4 nM and High:12.3 nM) and Afatinib (Low: 2.1 nM and High: 18.5 nM). Error bars and timepoints removed for visualization purposes. **b** Bcl-xL expression after afatinib treatment in concentrations 1 μM, 0.111 nM, 18.5 nM and 2.1 nM compared to DMSO control measured with high content microscopy. One-way ANOVA, $p < 0.0001$ for all conditions as compared to DMSO control. **c** Proposed schematic of interplay between EGFR, BIM and Bcl-xL. When inhibiting EGFR, BIM expression goes up but can still be inhibited by BcL-xL. When simultaneous inhibition of Bcl-xL and EGFR it creates a larger portion of free BIM which leads to apoptosis. Boxplot shows minimum, maximum and all points.

Supplementary Table 1: Clinical information for patient-derived cell models used in this paper

Case ID	Primary material	Cell Model	Diagnosis-histology	Stage	Indication for surgery	Operation	NACT-type	Postop treatment	Result	Relapse/progression	PFI (months)	Validation
OvCa_022	Tissue	cancer	HGS tubal cancer	3C	Interval debulking	Optimal	Car/Pac x3	Car/Pac/Bev	PR	Yes	5	panelSeq
OvCa_024	Tissue	cancer	LGSOC	4A	Interval debulking	Optimal	Car/Pac x3	Car/Pac/Bev	PR	Yes	5	panelSeq
OvCa_027	Tissue	cancer	HGSOC	4A	Primary debulking	Inoperable	0	Car/Pac/Bev	PR	Yes	1	panelSeq
OvCa_030	Ascites	cancer	HGS tubal cancer	3C	Primary debulking	Inoperable	0	Car/Pac/Bev	PR	Yes	7	panelSeq
OvCa_030	Ascites	fibroblast	HGS tubal cancer	3C	Primary debulking	Inoperable	0	Car/Pac/Bev	PR	Yes	7	panelSeq
OvCa_038	Ascites	fibroblast	HGSOC	4A	Primary debulking	Radical	Car/Pac x3	Car/Pac/Bev	CR	No	n.a.	panelSeq

Definitions	
Car	Carboplatin
Pac	Paclitaxel
Bev	Bevacizumab
Car/Pac x3	Three rounds of carboplatin and paclitaxel

Supplementary Table 2: Drugs included in the OC drug repurposing library

DRUG_NAME	Minimum Concen	Maximum Concent	Vendor	Product no	Mechanism/Targets	Drug class	Highest phase/Approval status	InChI Key	ChEMBL ID
8-chloro-adenosine	5	50000	Tocris	4436	Nucleoside analog, RNA synthesis inhibitor	A. Conv. Chemo	Investigational (Ph 2)	MHDPPLULTMGBSI-UUOKFMHZSA-N	CHEMBL1333540
A-1331852	0.1	1000	Selleckchem	S7801	Bcl-XL inhibitor	G. Apoptotic modulator	Probe	QCQQONWEDCOTBV-JLFAVICQSA-N	CHEMBL3793424
Adavosertib	1	10000	Selleckchem	S1525	WEE1 inhibitor	B. Kinase inhibitor	Investigational (Ph 2)	BKWJAKQVGHWELA-UHFFFAOYSA-N	CHEMBL1976040
Afatinib	0.1	1000	Selleckchem	S1011	EGFR inhibitor	B. Kinase inhibitor	Approved	ULXXDDBFHOBEHA-CWDCEQMOSA-N	CHEMBL1173655, CHEMBL2105712
Anagrelide	1	10000	Selleckchem	S3172	PDE-3, PLA2 inhibitor	X. Other	Approved	OTBXOEAOVRKTNQ-UHFFFAOYSA-N	CHEMBL1200759, CHEMBL760
Azacitidine	1	10000	Selleckchem	S1782	Nucleoside analog DNA methyl transferase inhibitor	E. Differentiating/ epigenetic modifier	Approved	NMUSYJAQQFHJEW-KVTDHHQDSA-N	CHEMBL1489
AZD8055	1	10000	Selleckchem	S1555	mTOR inhibitor	B. Kinase inhibitor	Investigational (Ph 1)	KVLFRAWTRWDEDF-IRXDYDNUSA-N	CHEMBL1801204
Birabresib	1	10000	Selleckchem		BET family inhibitor	E. Differentiating/ epigenetic modifier	Investigational (Ph 2)	GNMUEVRJHCWKTO-FQEVSTJZSA-N	not in ChEMBL
Birinapant	1	10000	Selleckchem	S7015	IAPs, SMAC mimetic	G. Apoptotic modulator	Investigational (Ph 2)	PKWRMUKBEYJEIX-DXXQBUJASA-N	CHEMBL3039522
BMS-754807	1	10000	Selleckchem	S1124	IGF1R inhibitor	B. Kinase inhibitor	Investigational (Ph 2)	LQVXSNNAFNGRAH-QHCPKHFHSA-N	CHEMBL575448
Canertinib	0.2	2000	Selleckchem	S1019	pan-HER inhibitor	B. Kinase inhibitor	Investigational (Ph 3)	OMZCMEYTWSXEPZ-UHFFFAOYSA-N	CHEMBL31965,CHEMBL545315
Carboplatin	50	500000	Merck	C2538	Platinum-based antineoplastic agent	A. Conv. Chemo	Approved	CCQPAEQGAVNNIA-UHFFFAOYSA-N	CHEMBL1351,CHEMBL288376
Cisplatin	10	100000	Merck	232120	Platinum-based antineoplastic agent	A. Conv. Chemo	Approved	DLBNRDWREIUHLD-UHFFFAOYSA-L	CHEMBL11359,CHEMBL1386,CHEMBL2068237
Crenolanib	1	10000	Selleckchem	S2730	PDGFRA and PDGFRB inhibitor	B. Kinase inhibitor	Investigational (Ph 2)	DYNHJHQFHQTFTP-UHFFFAOYSA-N	CHEMBL2105728, CHEMBL2146086
Dactinomycin	0.1	1000	Tocris	1229	RNA and DNA synthesis inhibitor	A. Conv. Chemo	Approved	RJURFGZVJUQBHK-IIXSONLDSA-N	CHEMBL1554
Dactolisib	0.1	1000	Selleckchem	S1009	mTOR/(PI3K) inhibitor	B. Kinase inhibitor	Investigational (Ph 2)	JOGKUKXHTYWRGZ-UHFFFAOYSA-N	CHEMBL1879463, CHEMBL1911126
Dasatinib	0.1	1000	Selleckchem	S1021	Abl, Src, Kit, EphR... Inhibitor	B. Kinase inhibitor	Approved	ZBNZXTGUTAYRHI-UHFFFAOYSA-N	CHEMBL1421
Daunorubicin	1	10000	Selleckchem	S3035	Topoisomerase II inhibitor	A. Conv. Chemo	Approved	STQGQHZAVUOBTE-VGBVRHCVSA-N	CHEMBL1563,CHEMBL178,CHEMBL434442,CHEMBL1200475
Dexamethasone	1	10000	Selleckchem	S1322	Glucocorticoid, immunomodulatory agent	D. Immunomodulatory	Approved	UREBDLICKHMUKA-CXSFZGCWSA-N	CHEMBL1200637, CHEMBL384467,CHEMBL1201302
Digoxin	0.1	1000	Selleckchem	S4290	Cardiac glycoside	X. Other	Approved (non-oncology)	LTMHDMANZUZIPE-PUGKRICDSA-N	CHEMBL1751
Doxorubicin	0.1	1000	MedChemEx	HY-15143/CS	Topoisomerase II inhibitor	A. Conv. Chemo	Approved	AOJJSUZBOXZQNB-TZSSRYMLSA-N	CHEMBL53463,CHEMBL272706,CHEMBL359744,CHEMBL216371
Erastin	1	10000	Selleckchem		VDAC inhibitor, induces ferroptosis	H. Metabolic modifier	Probe	BKQFRNYHFIQEKN-UHFFFAOYSA-N	CHEMBL401989
Erdafitinib	0.1	1000	Selleckchem	S8401	FGFR inhibitor	B. Kinase inhibitor	Investigational (Ph 2)	OLAHOMJCDNXHFI-UHFFFAOYSA-N	CHEMBL3545376
Filanesib	0.1	1000	Gift		KSP/Eg5 inhibitor	I. Kinesin inhibitor	Investigational (Ph 2)	LLXISKGBWFTGEI-FQEVSTJZSA-N	CHEMBL2347655
Fostamatinib	1	10000	Selleckchem	S2625	Syk inhibitor	B. Kinase inhibitor	Investigational (Ph 2)	GKDRMWXFWHEQQT-UHFFFAOYSA-L	CHEMBL2103830
Gandotinib	1	10000	Selleckchem	S2179	JAK2 inhibitor	B. Kinase inhibitor	Investigational (Ph 2)	SQSZANZGUXWJEA-UHFFFAOYSA-N	CHEMBL2107823
Gedatolisib	0.2	2000	Selleckchem	S2628	PI3K/mTOR inhibitor	B. Kinase inhibitor	Investigational (Ph 2))	DWZAEMINVBZMHQ-UHFFFAOYSA-N	CHEMBL592445
Gemcitabine	0.1	1000	Selleckchem	S1714	Antimetabolite; Nucleoside analog	A. Conv. Chemo	Approved	SDUQYLNIPVEERB-QPPQHZFASA-O	CHEMBL888,CHEMBL1637

Golvatinib	1	10000	Selleckchem	S2859	MET, VEGFR2 inhibitor	B. Kinase inhibitor	Investigational (Ph 2)	UQRCJCNVNUFYDX-UHFFFAOYSA-N	CHEMBL3039525
Idasanutlin	1	10000	Selleckchem	S7205	p53-MDM2 inhibitor	G. Apoptotic modulator	Investigational (Ph 3)	TVTXCJFHQKSQQM-LJQIRTBHSA-N	CHEMBL2402737
Indibulin	1	10000	Tocris	3728	Mitoric inhibitor. Microtubule depolymerizer	A. Conv. Chemo	Investigational (Ph 2)	SOLIIYNRSAWTSQ-UHFFFAOYSA-N	CHEMBL49642
Ipatasertib	1	10000	Selleckchem	S2808	AKT inhibitor	B. Kinase inhibitor	Investigational (Ph 2)	GRZXWCHAXNAUHY-NSISKUIASA-N	CHEMBL2177390
Mepacrine	1	10000	Selleckchem	S5435	Unclear. PLA2 inhibitor. NF-kB inhibitor, p53 activator	X. Other	Approved	GPKJTRJOBQGKQK-UHFFFAOYSA-N	CHEMBL2105615, CHEMBL554190,CHEMBL7568,CHEMBL556980,CHEMBL546257
Midostaurin	1	10000	Merck	M1323-1MG	Broad TK (FLT3, KIT, RET, JAK, EGFR...) inhibitor	B. Kinase inhibitor	Approved (US)	BMGQWWVMWDBQGC-IIFHNQTCSA-N	CHEMBL2309609, CHEMBL608533
Milciclib	1	10000	Selleckchem	S2751	CDK2 inhibitor	B. Kinase inhibitor	Investigational (Ph 2)	RXZMYLDMFYNEIM-UHFFFAOYSA-N	CHEMBL564829
Mubritinib	1	1000	Selleckchem	S2216	HER2 inhibitor	B. Kinase inhibitor	Investigational (Ph 1)	ZTFBIUXIQYRUNT-MDWZMJQESA-N	CHEMBL1614707
Neratinib	0.2	2000	Selleckchem	S2150	HER2, EGFR inhibitor	B. Kinase inhibitor	Approved (US)	JWNPDZNEKVCWMY-VQHVLOKHSA-N	CHEMBL180022
NVP-LCL161	5	50000	Selleckchem		IAPs, SMAC mimetic	G. Apoptotic modulator	Investigational (Ph 2)	UFPFGVNKHCLJJO-SSKFGXFMSA-N	CHEMBL2431768
Olaparib	1	10000	Selleckchem	S1060	PARP inhibitor	E. Differentiating/ epigenetic modifier	Approved	FDLYAMZZIXQODN-UHFFFAOYSA-N	CHEMBL521686
Omacetaxine	1	10000	Selleckchem		Protein synthesis inhib (80 S ribosome)	A. Conv. Chemo	Approved	HYFHYPWGAURHIV-JFIAXGOJSA-N	CHEMBL46286
Omipalisib	0.1	1000	Selleckchem	S2658	PI3K/mTOR inhibitor	B. Kinase inhibitor	Investigational (Ph 1)	CGBJSGAELGCMKE-UHFFFAOYSA-N	CHEMBL1236962
OSU-03012	1	10000	Selleckchem	S1106	PDPK1 inhibitor	B. Kinase inhibitor	Investigational (Ph 1)	YULUCECVQOCQFQ-UHFFFAOYSA-N	CHEMBL1650595
Paclitaxel	0.1	1000	Selleckchem	S1150	Mitotic inhibitor, taxane microtubule stabilizer	A. Conv. Chemo	Approved	RCINICONZNJXQF-MZXODVADSA-N	CHEMBL428647,CHEMBL48,CHEMBL1429740
PF-00562271	1	10000	Selleckchem	S2672	FAK inhibitor	B. Kinase inhibitor	Investigational (Ph 1)	MZDKLVOWGIOKTN-UHFFFAOYSA-N	CHEMBL1084546
PF-03758309	1	10000	Selleckchem	S7094	PAK inhibitor	B. Kinase inhibitor	Investigational (Ph 1)	AYCPARAPKDAOEN-LJQANCHMSA-N	CHEMBL3128043
Poziotinib	0.1	1000	Selleckchem	S7358	pan-HER inhibitor	B. Kinase inhibitor	Investigational (Ph 2)	LPFWVDIFUFFKJU-UHFFFAOYSA-N	CHEMBL3545154
Prexasertib	1	10000	MedChemEx	HY-18174/CS	Chk1 inhibitor	B. Kinase inhibitor	Investigational (Ph 2)	DOTGPNHGTYJDEP-UHFFFAOYSA-N	CHEMBL3544911
Ribociclib	1	10000	Selleckchem	S7440	CDK4/6 inhibitor	B. Kinase inhibitor	Approved (US)	RHXHGRAEPCAFML-UHFFFAOYSA-N	not in ChEMBL
Sirolimus	0.01	100	Selleckchem	S1039	binds FKBP12, causes inhibition of mTORC1	C. Rapalog	Approved	QFJCIRLUMZQUOT-HPLJOQBZSA-N	CHEMBL413
SN-38	1	10000	Selleckchem	S4908	Active metabolite of irinotecan. Topoisomerase I inhibitor	A. Conv. Chemo	(Approved)	FJHBVJOVLFPMQE-QFIPXVFZSA-N	CHEMBL837
Temsirolimus	0.01	100	Selleckchem	S1044	binds FKBP12, causes inhibition of mTORC1	C. Rapalog	Approved	CBPNZQVSJQDFBE-FUXHJELOSA-N	CHEMBL1201182
Topotecan	1	10000	Selleckchem	S1231	Topoisomerase I inhibitor. Camptothecin analog	A. Conv. Chemo	Approved	UCFGDBYHRUNTLO-QHCPKHFHSA-N	CHEMBL1607,CHEMBL84
Trametinib	0.1	1000	Selleckchem	S2673	MEK1/2 inhibitor	B. Kinase inhibitor	Approved	LIRYPHYGHXZJBZ-UHFFFAOYSA-N	CHEMBL2103875, CHEMBL2105741
Triapine	1	10000	Selleckchem	S7470	ribonucleotide reductase inhibitor	H. Metabolic modifier	Investigational (Ph 2)	XMYKNCNAZKMVQN-NYYWCZLTSA-N	CHEMBL231616
Ulixertinib	1	10000	Selleckchem	S7854	ERK inhibitor	B. Kinase inhibitor	Investigational (Ph 2)		
Venetoclax	0.1	1000	Selleckchem	S8048	Bcl-2-selective inhibitor	G. Apoptotic modulator	Approved (US)	LQBVNQSMGBZMKD-UHFFFAOYSA-N	CHEMBL3137309
Vinorelbine	0.1	1000	Selleckchem	S4505	Mitotic inhibitor. Vinca alkaloid microtubule depolymerizer	A. Conv. Chemo	Approved	GBABOYUKABKIAF-IELIFDKJSA-N	CHEMBL1200655, CHEMBL607994
Vistusertib	1	10000	Selleckchem	S2783	mTOR inhibitor, ATP-competitive	B. Kinase inhibitor	Investigational (Ph 2)	JUSFANSTBFGBAF-IRXDYDNUSA-N	CHEMBL2325741, CHEMBL2336325

Supplementary Table 3: Drug sensitivity scores for the two patient-derived models OvCa_027 and OvCa_024 using the TMRM parameter

Drug Name	OvCa_024	OvCa_027
8-chloro-adenosine	4	2.5
A-1331852	23.3	34.3
Adavosertib	2.9	9.5
Afatinib	2	7.5
Anagrelide	0	0
Azacitidine	3.1	4.3
AZD8055	3.9	4.5
Birabresib	15.6	3.9
Birinapant	15.7	39
BMS-754807	18.8	16.1
Canertinib	4.8	0.6
Carboplatin	11	8.5
Cisplatin	8.6	8.3
Crenolanib	0	0
Dactinomycin	22.2	17.8
Dactolisib	0.5	0
Dasatinib	0	0
Daunorubicin	19.2	14.5
Dexamethasone	0	0.8
Digoxin	11.2	13
Doxorubicin	6.6	4.3
Erastin	0.4	7.6
Erdafitinib	0	0
Filanesib	16.6	11.6
Fostamatinib	3.9	4.1
Gandotinib	2	0
Gedatolisib	0	0.2
Gemcitabine	5.1	0
Golvatinib	0	6.3

Drug Name	OvCa_024	OvCa_027
Idasanutlin	0	0
Indibulin	16.7	0
Ipatasertib	4.1	6.9
Mepacrine	7.4	7.7
Midostaurin	7.5	8.6
Milciclib	0	0.1
Mubritinib	0	0
Neratinib	0.6	4
NVP-LCL161	10	
Olaparib	0	0
Omacetaxine	14.2	15
Omipalisib	11.9	14.1
OSU-03012	0	0
Paclitaxel	15.8	0
PF-00562271	8.1	11.2
PF-03758309	15	5.4
Poziotinib	4.6	0
Prexasertib	5	11.6
Ribociclib	0	0
Sirolimus	0	0
SN-38	24.4	36.4
Temsirolimus	0	1
Topotecan	13.4	18.3
Trametinib	0	7.4
Triapine	3.2	0
Ulixertinib	0.1	2
Venetoclax	0	0
Vinorelbine	16.3	0
Vistusertib	0.6	1.6

Supplementary Table 4: Clinical information for the samples used in the prospective study.

Case ID	Material assayed	Diagnosis-histology	Stage	Ca-125	Indication for surgery	Operation	NACT-type	Postop treatment	Result	Relapse/progression	PFI (months)
OvCa_025	Tissue	HGS peritoneal cancer	2B	81	Primary debulking	Radical	0	Lost to follow-up	n.a.	n.a.	n.a.
OvCa_026	Tissue	HGS endometrial cancer	4B	2560	Primary debulking	Radical	0	Car/Pac	CR	Yes	4
OvCa_030	Tissue	HGS tubal cancer	3C	2960	Primary debulking	Inoperable	0	Car/Pac/Bev	PR	Yes	7
OvCa_031	Tissue	HGSOC	4A	3130	Interval debulking	Optimal	Car/Pac x3	Car/Pac/Bev	PR	Yes	8
OvCa_037	Tissue	HGSOC	3C	1620	Primary debulking	Radical	0	Car/Pac/Bev	CR	Yes	18
OvCa_038	Tissue	HGS tubal cancer	3C	125	Primary debulking	Radical	0	Car/Pac	CR	Lost to follow-up	n.a.
OvCa_039	Ascites	HGSOC	3C	103	Primary debulking	Inoperable	0	Car/Pac	PR	Not yet	>22
OvCa_040	Ascites	Struma ovarii with papillary thyroid cancer	1C	1720	Primary surgery	Radical	0	Thyroid surgery and radio-iodine	CR	Not yet	n.a.
OvCa_041	Ascites	HGSOC	3C	864	Primary debulking	Radical	0	Car/Lipdox	CR	Not yet	>21
OvCa_042	Tissue/ascites	Recurrent MUCOC from 2018	1A (2018)	356	Relapse surgery	Inoperable	0	Carbo	P	DOD	0
OvCa_043	Ascites	HGS tubal cancer	3C	531	Primary debulking	Radical	0	Car/Pac-Car/Lipdox	CR	Yes	15
OvCa_044	Tisssue	LGSOC	3C	99	Primary debulking after diagnostic surgery	Radical	0	Car/Lipdox	CR	Yes	11
OvCa_045	Tissue/ascites	Recurrent LGSOC from 1991	3A (1991)	318	Relapse surgery	Optimal	0	Car/Pac/Bev	PR	Yes	12
OvCa_047	Tissue/ascites	HGS tubal cancer	4B	2320	Primary debulking	Radical	0	Car/Pac/Bev	CR	Not yet	>18
OvCa_050	Tissue/ascites	HGS tubal cancer	3C	1580	Primary debulking	Optimal	0	Car/Pac/Bev	CR	Not yet	>15
OvCa_053	Ascites	HGS tubal cancer	3C	498	Primary debulking	Optimal	0	Car/Pac	CR	Yes	6.5

Definitions	
Car	Carboplatin
Pac	Palitaxel
Bev	Bevacizumab
Car/Pac x3	Three rounds Carboplatin and Paclitaxel
CR	complete response
PR	partial response
P	progression
DOD	died of disease

Supplementary Table 5: Drug sensitivity scores for the prospective study

Drug Name	025T	026T	030T	031T	037T	038T	039A	040A	041A	042T	042A	043A	044T	045T	045A	047T	047A	050T	050A	053A
8-chloro-adenosine	4.3	0.2	0.1	0.7	1.7	5.1	0	0.5	0.8	0	2.5	0	0	0.1	0	0	1.4	2.2	4.4	4.1
A-1331852	7.4	1.5	0	13.9	7.6	20.7	0	4.1	2.4	10.2	9.5	20.6	8.2	10.6	0	13.2	11.4	12.9	11	24.3
Adavosertib	5.7	8	0	0	10.8	4.1	13.1	0	1.6	4	0	5.2	0	0	1.5	11.3	4.6	0	0.8	2.9
Afatinib	0	7.3	0.6	6.9	1.9	8.2	0	17	0	0	0	4.4	0	0	1	0	0.9	0	0	4.2
Anagrelide	0	0.2	0	1.6	0	0	1.1	5.4	0.1	2.3	0	0	0	2.4	2.9	0.5	0	0	0	7.2
Azacitidine	0	2.9	1.5	0	8.9	0	3.2	2.4	0	6.1	0.9	0	0.8	2.9	2.4	5.6	3.8	0	0	0.9
AZD8055				6.5	7	13.7	0.2	18.7	8.9	3.7	8.7	0	0	0	1.6	3.1	9.4	3	5	10.5
Birabresib	5.6	4.7	1.8	1.2	2.1	0	0	9.2	4.7	0	3.3	4.8	4.3	0.6	2.9	9.9	0	5.1	4.7	19.7
Birinapant	4.3	17.4	6.8	7.2	6.6	4.6	0	4.8	0	1.2	2	0	1.4	0.3	0.6	3	1.4	3.6	4.7	32.4
BMS-754807	4.9	0.1	3.3	7.1	2.6	0	0.6	0	2.5	2.9	1.7	0	0.3	0	0	0	6.1	2.1	0	6
Canertinib	6.2	7.7	0	1.8	0	1.7	0	4.4	0	0	0	0	0	0	0	0	0	0	2	12.1
Carboplatin	11	7.5	1.9	3	11.6	20.2	7.4	11.5	12.2	4.4	5.5	4.5	1.8	3.7	3.9	8.8	8.5	7	6.3	11.8
Cisplatin	4.3	4.7	0	0	14.1	12.4	15.9	9.8	15.6	14.1	11.3	8.2	5.6	1.3	1.2	10.6	13.1	13.2	11.9	15.2
Crenolanib	3.7		0	0	0.1	9.2	0.2	0	0	0	0	0	0	0	0	0	0	1.1	0	0
Dactinomycin				20.5	20	13.1	7.4	18.1	16.4	10	3.2	0.4	0.7	0.9	1.7	14.2	8	10.9	14.3	18.2
Dactolisib	3.6	0	0	2.9	0	2.2	0	6.9	0	0.8	0.4	0	0	0	0.6	0	0	0.2	2.2	2.8
Dasatinib	0	5.4	0	0	0	12.3	0	0	1.8	0	0	0	0	0	0	0	0	0	0	20.2
Daunorubicin				14.1	19.1	9.1	0	15.4	14.7	6.1	7.7	0.8	2.7	3.5	3.7	3.1	5.6	9.1	16.1	6.1
Dexamethasone	0	0.1	2.6	0	0	3.5	4.3	16.9	0	0	0	0	0	0	0	0	0	0	0	0
Digoxin	12.5	7.9	4.6	5.8	7.4	8	12		16.2	4.6	1.8	38.6	2.2	7.6	5.6	10.4	6.4	5.4	7	9.5
Doxorubicin	2.5	4.1	0	5.9	0.5	2.6	0	11.2	6.9	3	1.2	3.2	0.6	0.3	0.7	0	1.2	3.6	5.4	3.8
Erastin	0	8.5	0	0	0	0	0	0	0.1	0	0	0	0	0	0	0	0	0	0.1	0
Erdafitinib	0.4	0.4	0	0	0		0.3	11	3.6	0	2.6	0	0	0.1	0	0	0	0	3.3	9.2
Filanesib	0.5	15.8	0	14.9	2	13.6	4.3	3.4	0	1.8	0	0	0	0	0	7.5	2.3	0.4	0	0.1
Fostamatinib	3.9	5	0.3	3.2	2.8	22.5	0.5	3.7	2.4	1.4	1.7	0	0	0	0.2	0	2.1	4.5	0	3.7
Gandotinib	0.4	5.4	0	0.7	0	7.9	2	0	0	0	0.4	0	2.4	0.5	0	4	0	0	0.7	7
Gedatolisib	12.4	12.7	0	6.4	0.6	0	0	10.8	6.5	11.6	3.1	0	0	0	0	0.2	2.7	7.3	11.9	17.9
Gemcitabine	0	0	0	0	0.6	0	0	0	3.8	5.1	1.8	0	0	0	0	0	0	0	1.8	0.3
Golvatinib	0	0	0.1	4.4	7.1	1.1	0	14.1	10.4	0	3.2	1.3	1.1	0	0	0	0	1.1	1.1	8.7
Idasanutlin	0	7.6	0	0	0	5.4	4.9	0	0	0	0	0	0	0.7	0	0	0	0	0	0
Indibulin	0.2	13.1	0	0	3.3	0	0	0.2	0	0	0	0	0	0	1.1	0	0	3.3	2.7	0
Ipatasertib	0	0.1	0	6.1	5.2	5.9	2.4	9.4	0	3.6	0	0	0	0.6	1.2	0	0.6	0	1.7	2.4
Mepacrine	3.4	7	0	7.3	5	3.2	0	11.8	7.5	0	0.6	2	0	0	3.1	0.3	14.3	0	2.1	6.2
Midostaurin	8.1	19.1	0.2	4.1	3.9	7.4	0.8	4.8	1.8	1.6	1.5	0	0	0	1.5	0	2.5	1.6	0.7	11.2
Milciclib	5.6	2.1	0.4	2.4	5.8	0	0	8.2	9	0	1.3	7.5	0	0	0	6.7	1.9	2.2	0	7.5
Mubritinib	2.8	0	0.3	0	0	0	0	13.6	3.7	7.7	4.6	0	7	5.7	2.8	11.6	32.2	1.7	0	0
Neratinib	11.5	0	0	4	0	0	0	1	2.9	0	0	0	0	0	0	0	0.9	0	0	1.3
NVP-LCL161	1.8	20.3	11.9	4.7	19	8.5	0.1	0.7	0	0	0	0	0	0	0	0	0	0	0	18.5
Olaparib	0	0.4	2.8	0	0.4	0	0	0	2.7	0	0	0	2.5	0.1	0.1	0	1.8	0	0	0
Omacetaxine	16.2	16.3	10.4	15.5	6.1	10.4	0	10.9	11.8	4.3	3.4	0	3.5	2.9	7.5	8.5	12	13.3	11.6	17.7
Omipalisib	11.2	15.1	4.2	14.9	10.7	17.9	4.2	15.3	17.7	8.2	7.8	0	0	0	6.6	11.4	10	9.7	19	20.4
OSU-03012	0	5.7	0.2	0	0	0	0	9	0.2	0	0	0	0	0	0	0	0	0	0	2.8
Paclitaxel	4.2	20	0	19	0	6.8	0	2.5	0	0	0.9	0	2.8	0	0	0	0	0.3	5.5	0
PF-00562271	4.9	3.5	3.3	6.9	0	2.5	5.7	16.2	5.7	1.3	2.7	0	0	0	0	3.9	1.4	4.6	3.5	4.9
PF-03758309	5.3	12.4	5.6	6.1	6.4	9.5	1.9	3.8	8.6	1.5	3.1	0.4	2.5	0	0.6	0.3	5.4	4.2	6.1	9.7
Poziotinib	2.8	4.1	0	1.4	0.1	0	1.4	8.2	0	0	4.6	0.2	0	0	4.5	0	0	0.3	0	1
Prexasertib	3.5	24	2.8	7.3	17.8	0	0	19.9	3.4	0	3	0	1	0	0	0	1.4	3.6	0	19
Ribociclib	0	0	0	0.4	0	0	0	5.8	1.1	0	0	0	0	0	0.2	0.4	0	0	0	14.4
Sirolimus	4.6	0	2.9	0	0	0.2	3.1	1.2	1	0	2.5	0	0	0	0	0	0	1.8	2.6	0
SN-38				10.3	3.4	12	0	16.2	8.2	4.8	10.7	31	0	4.8	1.7	11.6	0	11.1	7.9	14.7
Temsirolimus	1.6		0	0	0	0	2.4	14.6	0	0	0	0	0	0	0	0	0	0	1.2	0
Topotecan	6.2	12.4	4.2	7	11.3	18.5	1.7	17.3	4.7	11.9	9.9	0	0	0	0	6.5	2.4	4.5	4.7	8.7
Trametinib	5.4	1.5	0	0.6	0.7	1.2	0	8.6	9.1	3.9	3.6	0.2	0	0	0.4	0	0	3.9	3.3	8.9
Triapine	1.2	0.3	0	0.3	0.2	2.4	1.4	3.8	1.6	1.5	1.1	0	3.3	0.4	0	7.2	18.6	1.1	2.5	1.3
Ulixertinib	2	1.2	0	1.6	8.9	0	4.6	2.1	6	0	1.7	1	0	0	0	0	0	0	3.3	22.1
Venetoclax	0	1.5	0	0.1	2.4	0	0	0	0	3	0	0	0.5	0.9	0	1	0	0	0	8.9
Vinorelbine	2.5	14.5	0	4.1	6		8.1	6.6	0	0	0	0	0	0	0.8	5.2	0.4	2.6	1.9	1.1
Vistusertib	7.8	4.7	0	4.3	5.7	18.8	6.5	11.3	2.3	0	4.7	0	0	0	0.1	0	6.1	1.4	5.9	9.6

Supplementary Table 6: Combinations tested for each patient and the ZIP synergy score

Sample				
038T	Combinations	Carboplatin - A-1331852	Carboplatin - Dactinomycin	Carboplatin - A1155463
	Drug mechanisms or target	DNA damage - Bcl-xL	DNA damage - chemotherapy	DNA damage - Bcl-xL
	ZIP synergy score	9.18	-1.9	5.37
	Most synergistic area score	18.6	3.24	14.71
040A	Combinations	Carboplatin - A-1331852	Carboplatin - Birinapant	A-1155463 - Birinapant
	Drug mechanisms or target	DNA damage - Bcl-xL	DNA damage - XIAP	DNA damage - Bcl-xL
	ZIP synergy score	-2.89	-0.4	-1.54
	Most synergistic area score	-1	1.86	6.92
042T	Combinations	Carboplatin - A-1331852	Carboplatin - Selumetinib	A-1331852 - Selumetinib
	Drug mechanisms or target	DNA damage - Bcl-xL	DNA damage - MEK	Bcl-xL - MEK
	ZIP synergy score	-0.87	-0.10	11.58
	Most synergistic area score	2.09	1.34	16.08
043A	Combinations	Carboplatin - A-1331852	Carboplatin - Afatinib	A-1331852 - Afatinib
	Drug mechanisms or target	DNA damage - Bcl-xL	DNA damage - EGFR	Bcl-xL - EGFR
	ZIP synergy score	-0.2	3.25	12.38
	Most synergistic area score	2.53	6.22	21.12
044T	Combinations	Carboplatin - A-1331852	Carboplatin - Dactolisib	A-1331852 - Dactolisib
	Drug mechanisms or target	DNA damage - Bcl-xL	DNA damage - MTOR	Bcl-xL - MTOR
	ZIP synergy score	7.73	-8.73	-0.98
	Most synergistic area score	11.15	-4.54	2.54
045T	Combinations	Carboplatin - A-1331852	Carboplatin - Prexasertib	A-1331852 - Prexasertib
	Drug mechanisms or target	DNA damage - Bcl-xL	DNA damage - CHEK1	Bcl-xL - CHEK1
	ZIP synergy score	-6.22	-6.29	0.51
	Most synergistic area score	-1.74	-1.58	4.21
050T	Combinations	Carboplatin - A-1331852	Carboplatin - Omacetaxine	A-1331852 - Omacetaxine
	Drug mechanisms or target	DNA damage - Bcl-xL	DNA damage - protein synthesis	Bcl-xL - protein synthesis
	ZIP synergy score	-0.74	-0.73	14.32
	Most synergistic area score	4.11	4.5	38.07
050A	Combinations	Carboplatin - A-1331852	Carboplatin - Omacetaxine	A-1331852 - Omacetaxine
	Drug mechanisms or target	DNA damage - Bcl-xL	DNA damage - protein synthesis	Bcl-xL - protein synthesis
	ZIP synergy score	10.9	0.21	23.5
	Most synergistic area score	20.17	4.03	43.37
053A	Combinations	Carboplatin - A-1331852	Carboplatin - Birinapant	A-1331852 - Birinapant
	Drug mechanisms or target	DNA damage - Bcl-xL	DNA damage - XIAP	Bcl-xL - XIAP
	ZIP synergy score	7.19	1.05	-5.75
	Most synergistic area score	8.16	6.2	0.29
